# Bilayer vascular grafts separately loaded with sodium copper chlorophyllin and keratin-based hydrogen sulfide donor with pro-endothelialization, anti-thrombogenicity, anti-inflammation, and anti-calcification properties

**DOI:** 10.1016/j.bioactmat.2025.11.001

**Published:** 2025-11-13

**Authors:** Yu Sun, Shunqi Hu, Lijuan Wang, Fubang Liang, Zeyi Zhou, Yuyuan Zhang, Yanjun Pan, Jian Shen, Meng Yin, Jiang Yuan

**Affiliations:** a Jiangsu Collaborative Innovation Center of Biomedical Functional Materials, Department of Materials Science and Engineering, School of Chemistry and Materials Science, Nanjing Normal University, Nanjing, 210023, PR China; b Department of Cardiothoracic Surgery, Shanghai Children's Medical Center, School of Medicine, Shanghai Jiao Tong University, 1678 Dong Fang Road, Shanghai, 200127, PR China; c Department of Cardiothoracic Surgery, Nanjing Drum Tower Hospital, Affiliated Hospital of Medical School, Nanjing University, Nanjing, PR China; d Wisdom Lake Academy of Pharmacy, Xi'an Jiaotong-Liverpool University, Suzhou, PR China

**Keywords:** Nitric oxide, Hydrogen sulfide, Small-diameter vascular grafts, Endothelialization

## Abstract

The clinical utility of small-diameter vascular grafts (SDVGs) remains limited due to thrombosis, inflammation, and intimal hyperplasia, which compromise long-term patency. Mimicking the structures and functions of blood vessels, bilayer SDVGs with gradient pore sizes were fabricated, thereby preventing the infiltration of vascular smooth muscle cells (VSMCs) into the inner layer. Additionally, sodium copper chlorophyllin (SCC) was embedded in the inner layer, potentially generating NO from endogenous donors in the blood and regulating vascular cells. Keratin-based H_2_S donor of KSN was synthesized and then electrospun with poly(L-lactide-co-ε-caprolactone) (PLCL) to serve as the outer layer of the grafts. The bilayer grafts promoted rapid endothelialization by selectively enhancing the adhesion, proliferation, and migration of vascular endothelial cells (VECs) while inhibiting those of VSMCs. More importantly, the released NO and H_2_S synergistically enhanced the anti-thrombotic, anti-inflammatory, and anti-calcification properties of the grafts. Furthermore, the bilayer grafts maintained the contractile phenotype of VSMCs and polarized macrophages toward the M2 phenotype. The grafts achieved patency with negligible intimal hyperplasia and calcification in the rat abdominal aorta replacement models for 1 month of implantation. The grafts modulated VECs via the PI3K-AKT signaling pathway, focal adhesion, apoptosis, and regulation of actin cytoskeleton, while regulating VSMCs through gap junction, ECM-receptor interaction, adherens junction, focal adhesion, and PI3K-AKT signaling pathway. These bilayer grafts with rapid endothelialization, antithrombogenicity, anti-inflammation, and anti-calcification properties are promising candidates for tissue-engineered SDVGs.

## Highlights

• Novel keratin-based donor of H_2_S was synthesized and subsequently electrospun to act as the outer layer of bilayer grafts to prolong the release of H_2_S.
• Biosafety sodium copper chlorophyllin (SCC) was incorporated and subsequently electrospun to act as the inner layer of bilayer grafts to catalytic generation of NO.
• Bilayer grafts promoted EC proliferation while inhibiting SMC proliferation.
• Bilayer grafts accelerated endothelialization without intimal hyperplasia, thrombosis, and calcification in vivo.

## Graphical abstract

**Figure:**
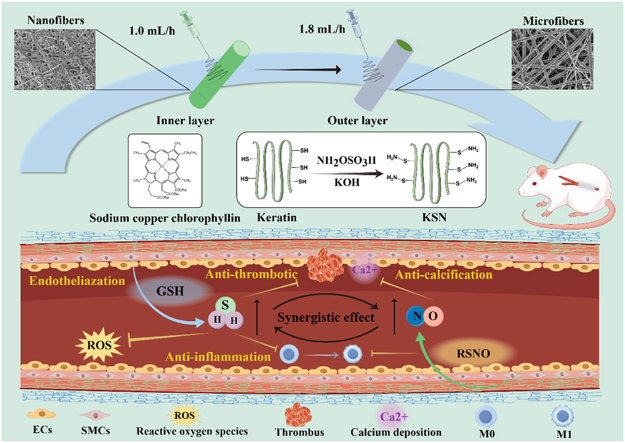

## 1 Introduction

The increasing incidence of diseases such as atherosclerosis, arterial hemangioma, vascular complications of diabetes, and vascular embolism leads to a growing demand for vascular transplantation [1]. Tissue-engineered vascular grafts (TEVGs) provide a regenerative solution for the shortage of autologous vessels. However, small-diameter vascular grafts (SDVGs< 6 mm) are associated with a risk of acute thrombosis, intimal hyperplasia, and calcification, ultimately leading to vascular occlusion and transplant failure [2]. This predicament is linked to the pathological cascade reaction of vascular remodeling. Delayed endothelialization exposes the subcutaneous matrix, triggering platelet aggregation and inflammation, promoting vascular smooth muscle cells (VSMC) transformation into the synthetic phenotype, followed by their proliferation and migration to the intima, leading to hyperplasia [[3], [4], [5]]. Notably, this pathological remodeling drives restenosis [6], bypass failure [7], accelerated atherosclerosis [8] after angioplasty, and forms a vicious cycle with graft calcification. Intimal calcification in vascular grafts arises from lipid infiltration and chronic inflammation, while the osteogenic differentiation of VSMCs drives medial calcification. Consequently, developing SDVGs that fulfill the clinical transplantation requirements remains an urgent challenge.

Natural blood vessels are hierarchically structured from the nanoscale to the macroscale with three concentric layers: intima, media, and adventitia. Each layer exhibits distinct differences in composition, mechanical properties, and thickness distribution [9,10]. Vascular endothelial cells (VECs) in the intima are aligned in an orderly manner along the longitudinal axis of blood vessels, forming an antithrombotic interface that directly contacts the blood. VSMCs in the media are predominantly distributed peripherally and dynamically contract to regulate hemodynamics. Notably, lipophilic nitric oxide (NO) secreted by VECs in the intima possesses multifaceted regulatory functions, including modulation of vascular tension, promotion of endothelial repair, inhibition of excessive proliferation of VSMCs, antagonism of platelet activation, and regulation of inflammatory responses [11,12]. Moreover, hydrogen sulfide (H_2_S) secreted by VSMCs exhibits biological activities analogous to NO within the cardiovascular system, including anti-inflammatory, antioxidant, vasodilatory, anti-fibrotic, and myocardial protective effects [13,14]. Further studies have demonstrated that NO and H_2_S can synergistically act as smooth muscle tone regulators, enhancing cardiovascular protection [15]. However, there remains a paucity of reports on functional materials that promote vascular regeneration based on the synergistic effects of dual-gas molecules. Herein, mimicking the natural blood vessel, we aim to fabricate vascular grafts capable of continuously releasing NO and H_2_S. The grafts can intervene in pathological processes, such as intimal hyperplasia and ectopic calcification, through the synergistic effect of dual-gas signaling molecules. To achieve this goal, the grafts must meet the microenvironmental requirements for the functional reconstruction of vascular cells. Electrospinning technology enables the precise control of fiber diameter and porosity, allowing for a 3D porous scaffold that mimics the extracellular matrix (ECM) and provides a biomimetic interface for cell adhesion and directional differentiation [16]. Additionally, the flow rate regulation can reconstruct the hierarchical structure of vascular grafts, resulting in bilayers with gradient pore sizes, an inner dense layer that simulates the intima, and an outer loose layer that promotes tissue integration.

**Fig. 1:**
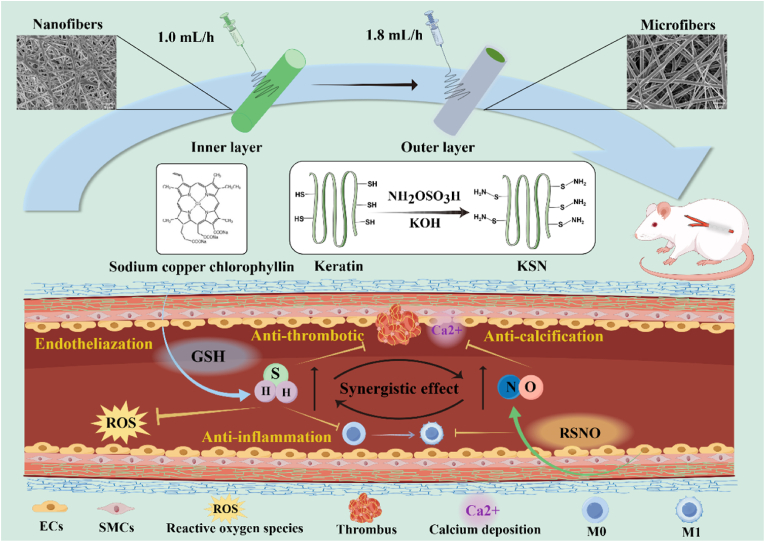
Schematic fabrication of PLCL/KSN//PLCL/SCC bilayer grafts. The SCC and KSN were introduced and synthesized for the first time. The pore sizes of bilayers were controlled by adjusting the flow rates during electrospinning to reduce the SMC infiltration into the inner layer. The bilayer grafts exhibited pro-endothelialization, antithrombogenicity, anti-inflammation, and anti-calcification properties.

Sodium copper chlorophyll (SCC) is an approved food colorant in China, the United States, and the European Union (EU), and thus has high biosafety [17]. Additionally, SCC has been demonstrated to possess potential antioxidant and antimicrobial properties [18]. SCC is proposed to catalyze the release of NO from endogenous donors in blood. Herein, SCC was introduced and electrospun with poly(L-lactide-co-ε-caprolactone) (PLCL) to serve as the inner layer for the first time (Fig. 1). In addition, a novel keratin-based hydrogen sulfide donor (KSN) was synthesized and coelectrospun with PLCL to act as the outer layer. The bilayer grafts with gradient pore sizes were prepared by adjusting the flow rate during electrospinning. The morphology, chemical composition, mechanical properties, hydrophilicity, and degradability of the grafts were evaluated. Cell migration, proliferation, polarization, and blood compatibility were investigated. Finally, the tubular bilayer grafts were implanted in a rat model of abdominal aorta replacement to evaluate vascular regeneration. RNA sequencing was conducted to elucidate the potential mechanism of vascular tissue remodeling.

## 2 Results

### 2.1 Preparation and characterization of PLCL/SCC and PLCL/KSN mats

#### 2.1.1 Synthesis and characterization of KSN

**Fig. 2:**
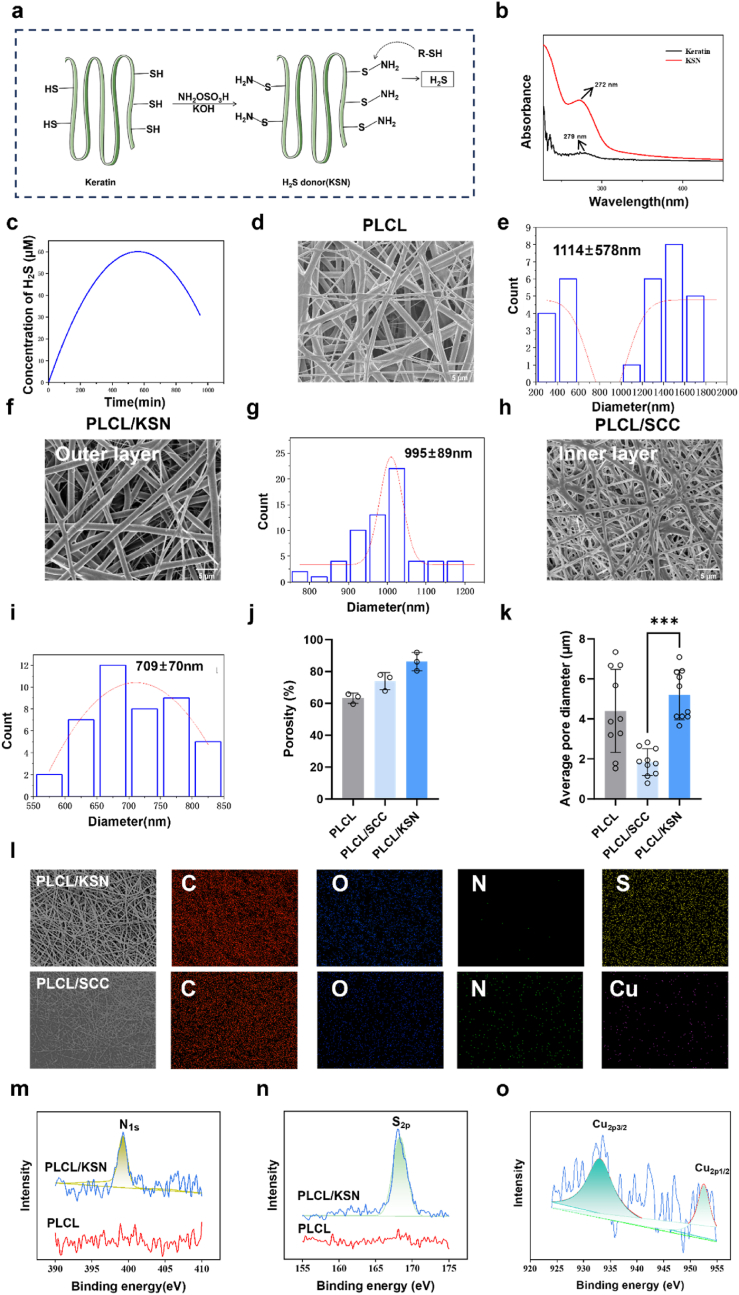
(a) Schematic synthesis of KSN and the release mechanism of H_2_S; (b) UV–Vis spectra of the keratin and KSN; (c) Cumulative release of H_2_S from KSN in the presence of GSH; (d–i) SEM images and diameter distribution of PLCL, PLCL/KSN, and PLCL/SCC fibers; (j) Porosity of PLCL/KSN and PLCL/SCC mats; (k) Pore diameters of PLCL/KSN and PLCL/SCC mats; (l) EDS mapping of C, O, N, S, and Cu elements in PLCL/KSN, and PLCL/SCC mats; (m) N_1s_ high-resolution XPS spectra of PLCL and PLCL/KSN mats; (n) S_2p_ high-resolution XPS spectra of PLCL and PLCL/KSN mats; (o) Cu_2p_ high-resolution XPS spectra of PLCL/SCC mats. Data are expressed as means ± SD. ∗p < 0.05, ∗∗p < 0.01, and ∗∗∗p < 0.001.

The H_2_S donor of KSN was synthesized and characterized (Fig. 2a). Compared to keratin, the maximum peak of KSN for UV spectroscopy shifted from 272 nm to 279 nm (Fig. 2b). H_2_S release results also indicated that KSN was synthesized successfully (Fig. 2c). FT-IR spectroscopy was also used to test, but there were no substantive differences between keratin and KSN (Fig. S1).

#### 2.1.2 Preparation and characterization of mats

The fiber diameters were controlled by adjusting the flow rates during electrospinning. The morphologies of PLCL, PLCL/KSN, and PLCL/SCC fibers were observed using scanning electron microscopy (SEM) (Fig. 2d,f, h). Diameters of PLCL, PLCL/KSN, and PLCL/SCC fibers are 1114 ± 578, 995 ± 89, and 709 ± 70 nm, respectively (Fig. 2e,g, i). The fiber diameters gradually decreased with the addition of KSN and SCC. The porosities of PLCL/KSN and PLCL/SCC mats are 86.2 % and 74.0 % with no significant differences (Fig. 2j). However, the pore sizes of the two mats are significantly different. The average pore size for PLCL/KSN mats is 5.19 ± 1.24 μm. In contrast, that of PLCL/SCC mats is 1.85 ± 0.67 μm (Fig. 2k). The energy-dispersive spectroscopy (EDS) images reveal the presence of carbon (C), oxygen (O), sulfur (S), and nitrogen (N) atoms in PLCL/KSN mats (Fig. 2i). As expected, Cu atom was detected in PLCL/SCC mats (Fig. 2l). X-ray photoelectron spectroscopy (XPS) demonstrated that the N and S elements are primarily derived from keratin with peaks of N_1s_ (400.0 eV), S_2p1/2_ (162.6 eV), and S_2p3/2_ (167.8 eV). The two Cu_2p_ weak peaks at 932.1 eV and 951.5 eV, corresponding to Cu_2p3/2_ and Cu_2p1/2_, are attributed to Cu^+^ and Cu^2+^, respectively (Fig. 2m–o, Fig.S2). These results confirm the successful and reliable fabrication of the PLCL/KSN and PLCL/SCC mats. The release curve of copper ions from PLCL/SCC mats is shown in Fig. S3, indicating that copper ions can be released sustainably for 7 d. The biomedical applications of PLCL are limited due to its hydrophobic property. In this study, introducing KSN and SCC improved the hydrophilicity of PLCL. The water contact angles were reduced from 118.5° to 55.1° and 24.8°, respectively (Fig. S4). The improved hydrophilicity would improve the biocompatibility of vascular grafts.

#### 2.1.3 The releases of NO and H_2_S

**Fig. 3:**
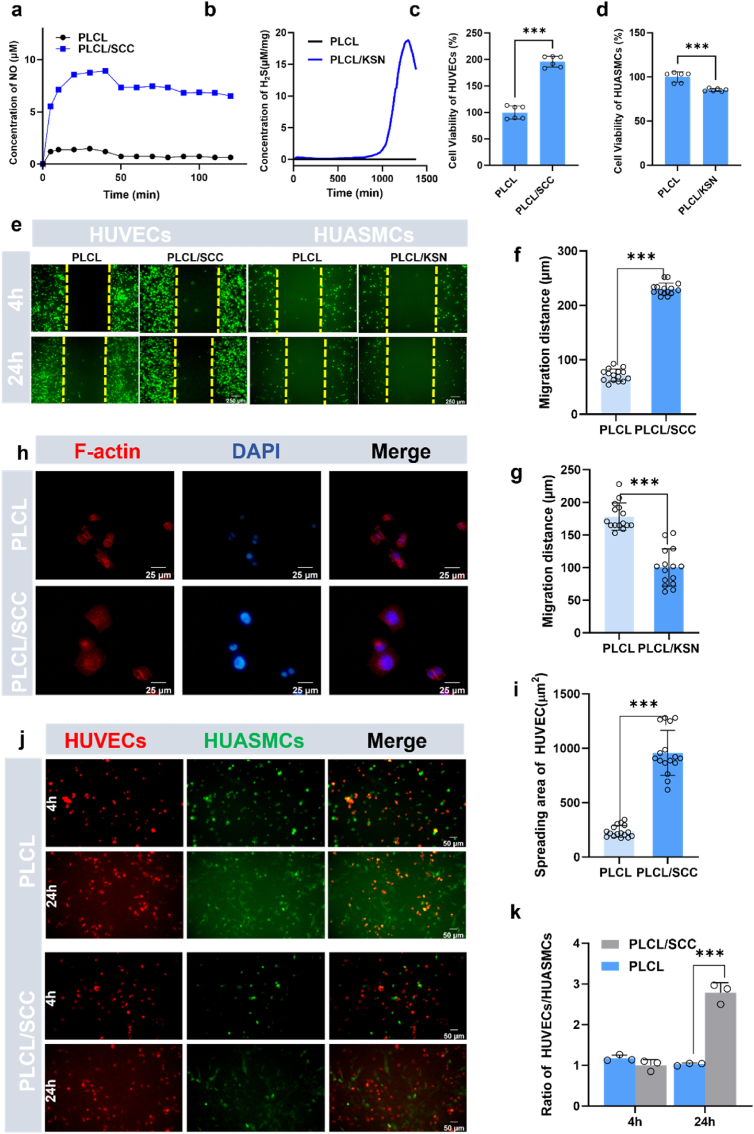
(a) The regeneration of NO from PLCL and PLCL/SCC mats in the presence of GSNO (50 μM); (b) Cumulative release of H_2_S from PLCL and PLCL/KSN mats in the presence of GSH (200 μM); (c) Cell viability of HUVECs on PLCL and PLCL/SCC mats in the presence of GSNO (50 μM); (d) Cell viability of HUASMCs on PLCL and PLCL/KSN mats in the presence of GSH (200 μM); (e) Migration images of HUVECs on PLCL/SCC mats in the presence of GSNO (50 μM) and HUASMCs on the PLCL/KSN mats in the presence of GSH(200 μM); (f) Migration distance of HUVECs on PLCL and PLCL/SCC mats; (g) Migration distance of HUASMCs on PLCL and PLCL/KSN mats; (h) Fluorescent images of the cytoskeleton of HUVECs on PLCL and PLCL/SCC mats; (i) Statistical cell spread area of HUVECs on PLCL and PLCL/SCC mats; (j) Fluorescence images of co-cultured HUVECs and HUASMCs on PLCL and PLCL/SCC mats; (k) Cell ratios of HUVECs and HUASMCs at 4 h and 24 h on PLCL/SCC mats. Data are expressed as means ± SD. ∗p < 0.05, ∗∗p < 0.01, and ∗∗∗p < 0.001.

The catalytic generation of NO from endogenous donors, constantly produced in the bloodstream, is a promising approach. Copper ions are capable of catalyzing S-nitrosothiol decomposition to generate NO [19,20]. In this study, we evaluated the catalytic generation of NO in the presence of GSNO using the Griess reagent. The results show that PLCL/SCC mats could continuously generate NO for 120 min. At the same time, there was no NO release in PLCL mats (Fig. 3a). The PLCL/SCC layer, acting as an inner layer of bilayer grafts, can induce the in-situ release of NO from endogenous donors in blood, enabling long-lasting NO production.

Keratin-based donor of KSN can release H_2_S in response to thiols, such as glutathione (GSH) or cysteine [21]. In this study, KSN was embedded in PLCL fibers, and H_2_S began to be released only after 1000 min (Fig. 3b). This design suggests that PLCL/KSN mats are envisioned to release H_2_S in line with the graft degradation. In summary, the grafts can exert protective potential in vascular regeneration by releasing NO and H_2_S from the inner and outer layers.

### 2.2 Pro-endothelialization and anti-intimal hyperplasia of mats

#### 2.2.1 The effect of NO release on HUVECs for PLCL/SCC mats

The media and intima of blood vessels are primarily composed of VSMCs and VECs. To understand the bilayer graft functionality, we characterized the regulation of HUASMCs on PLCL/KSN mats, which were used as the outer layer of the graft, and the HUVEC growth behavior on PLCL/SCC mats, which acted as the inner layer of the graft.

The viability of HUVECs on PLCL and PLCL/SCC mats in the presence of GSNO is illustrated (Fig. 3c). Results show that PLCL/SCC mats promoted the growth and proliferation of HUVECs due to the catalytic generation of NO. The generation of NO also facilitates the migration of HUVECs (Fig. 3e). It was measured that the migration distance on PLCL/SCC mats (ca. 230 μm) was significantly higher than that on PLCL mats (ca. 72 μm, p < 0.001) (Fig. 3f). This rapid cell migration of HUVECs from the anastomosis site to the center of the mats could promote endothelialization.

The cytoskeleton is essential for maintaining endothelial structural integrity, vascular permeability barrier, and cell signaling. The cytoskeleton is a three-dimensional network of microtubules, F-actin, and intermediate filaments. F-actin, labeled with phalloidin, is distributed in a quadrilateral two-dimensional network structure on PLCL/SCC mats (Fig. 3h). The filaments exhibit a highly ordered, fibrous structure. However, the F-actin of HUVECs on PLCL mats exhibits an atrophied structure. The cytoskeleton spreading area of HUVECs on PLCL/SCC mats is significantly larger than that on PLCL mats (Fig. 3i). F-actin is involved in cell division and migration and maintains the shape and structure of cells. Thus, the results indicate that PLCL/SCC, as the inner layer of the grafts, can preserve the cytoskeleton of HUVECs and potentially promote endothelialization.

HUVECs and HUASMCs were co-cultured on PLCL/SCC mats to study the cell selectivity of materials. At the initial 4 h, the ratios of HUVECs and HUASMCs on PLCL and PLCL/SCC are close to 1 (Fig. 3j). However, after 24 h, the ratio of HUVECs to HUASMCs on PLCL/SCC mats increased to approximately 2.97, while the ratio on PLCL mats was 1.04 (Fig. 3k). This suggests that PLCL/SCC mats are more selective towards HUVECs than HUASMCs, making them an ideal choice as the inner layer of the grafts. Results indicated that PLCL/SCC mats exhibit good biocompatibility and can facilitate endothelialization as the inner layer of the grafts.

#### 2.2.2 The effect of H_2_S on HUASMCs for PLCL/KSN mats

Migration of VSMCs into the intima is a major cause of intimal hyperplasia [22]. Similarly, we measured the cell viability, proliferation, and migration of HUASMCs on PLCL and PLCL/KSN mats. Compared with PLCL, PLCL/KSN mats slightly hindered the proliferation of HUASMCs in the presence of GSH due to the release of H_2_S(Fig. 3d). The release of H_2_S also inhibited the excessive migration of HUASMCs (Fig. 3g). The migration distance of HUASMCs on PLCL/KSN mats dropped from ca. 178 μm to ca. 100 μm in the presence of GSH. These results demonstrate that PLCL/KSN mats, as an outer layer of grafts, can prevent neointimal hyperplasia and restenosis in vascular regeneration.

#### 2.2.3 Synergistic effects of NO and H_2_S on the viability of vascular cells on bilayer mats

**Fig. 4:**
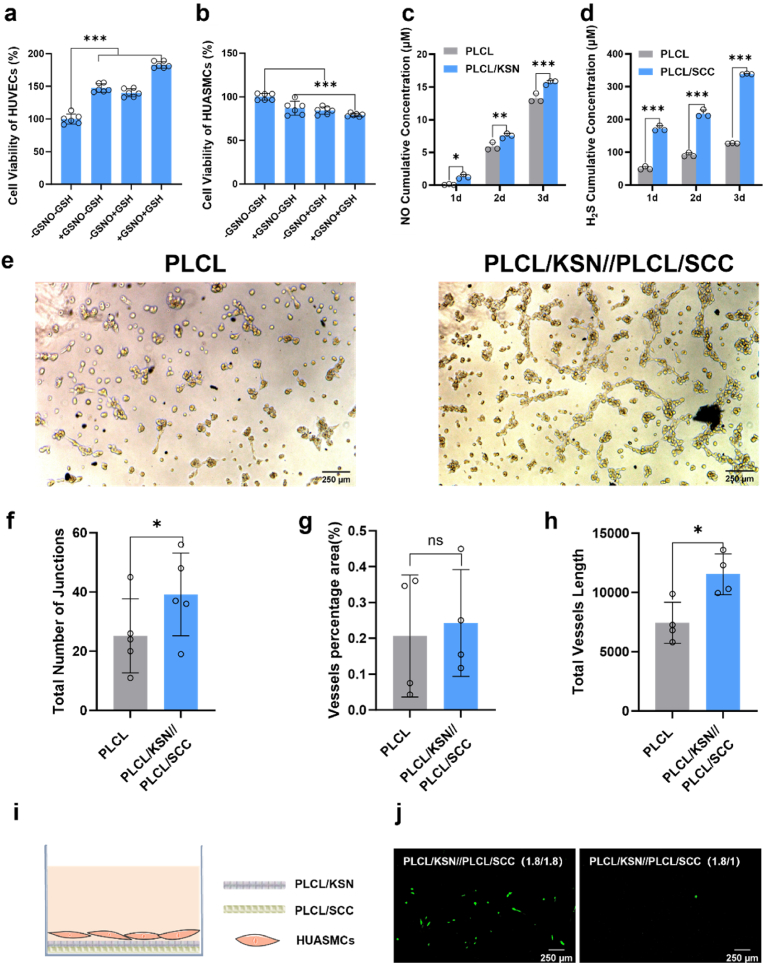
(a) Cell viability of HUVECs cultured on bilayer mats for 3 d (+200 μM GSH, +50 μM GSNO); (b) Cell viability of HUASMCs cultured on bilayer mats for 3 d (+200 μM GSH, +50 μM GSNO); (c) Cumulative NO secretion from HUVECs cultured on PLCL/KSN mats from day 1–3 (+50 μM GSNO); (d) Cumulative H_2_S secretion from HUASMCs cultured on PLCL/SCC mats from day 1–3 (+200 μM GSH); (e) Tube formation assay of HUVECs incubated with PLCL and bilayer mats for 6 h; (f–h) Quantitative statistic results of junctions, vessel area percentage, and total vessel length incubated with PLCL and bilayer mats for 6 h; (i) Schematic illustration of the incubation of HUASMCs on bilayer mats; (j) Fluorescent image of HUASMCs infiltrating from outer layer(flow rates are 1.8 or 1.0 mL/h during electrospinning) to inner layer(flow rate is 1.0 mL/h during electrospinning). Data are expressed as means ± SD. ∗p < 0.05, ∗∗p < 0.01, and ∗∗∗p < 0.001.

CCK-8 kits were used to measure the cell viability of HUVECs and HUASMCs on PLCL/KSN//PLCL/SCC bilayer mats. Compared with bilayer mats without GSNO and GSH, the presence of GSNO or GSH broadly promoted the proliferation of HUVECs, while the proliferation of HUASMCs was suppressed (Fig. 4a,b). Quantitatively, the presence of GSNO increased the viability of HUVECs to 150 % and inhibited the HUASMCs to 87 %. Similarly, the presence of GSH promoted the HUVECs cell proliferation to 140 % and decreased the viability of HUASMCs to 84 %. Furthermore, the viability of HUVECs was enhanced to 200 % in the presence of both GSNO and GSH, while the viability of HUASMCs was inhibited and decreased to 75 %. The proliferation of HUVECs and inhibition of HUSAMCs are believed to be contributed by the release of NO and H_2_S. The two-factor ANOVA analysis shows that NO and H_2_S significantly affect the cell behavior. The interaction effect between NO and H_2_S is significant (p < 0.05), suggesting that bilayer grafts could exert a synergistic effect of NO and H_2_S, accelerating vascular regeneration.

To verify the synergistic effects of NO and H_2_S and the interaction between NO and H_2_S, the impacts of H_2_S released from PLCL/KSN mats on the secretion and expression of NO in HUVECs and NO released from PLCL/SCC mats on the secretion and expression of H_2_S in HUASMCs were detected, respectively. As shown in Fig. 4c, the release of H_2_S from PLCL/KSN mats promotes HUVECs to secrete more NO over time. Exogenous H_2_S can positively regulate the eNOS signaling pathway and upregulate the expression of eNOS [23]. The secretion of NO is a crucial function of endothelial cells, and the release of H_2_S can help restore endothelial cell function and accelerate endothelialization. The expression of H_2_S by HUASMCs significantly increased in the presence of GSNO from PLCL/SCC mats, indicating that the released NO promoted the secretion of H_2_S in HUASMCs (Fig. 4d). This is mainly attributed to the fact that NO can directly increase the transcription level of CSE in SMCs, upregulate the expression of (cystathionine-γ-1yase)CSE, and further increase the production of H_2_S [24]. Additionally, NO can directly act on the free sulfhydryl group contained in the nitroso group of the CSE protein, thereby increasing CSE activity. The results suggested that NO and H_2_S not only directly maintain the normal function of HUASMCs and HUVECs but also indirectly regulate cell growth by promoting the expression of others. The interplay between H_2_S and NO pathways plays a crucial role in the vasoactive function during vascular lesions [15]. Thus, it was speculated that NO and H_2_S not only have regulatory effects on vascular cells alone but also promote the expression of the other through the corresponding signaling pathway.

#### 2.2.4 Angiogenesis capacity of bilayer mats on HUVECs

The main components of Matrigel are laminin, type IV collagen, and various growth factors [25,26]. In this study, Matrigel supports the angiogenesis of the grafts in vitro. The networks formed by HUVECs on bilayer mats were significantly more pronounced than those on PLCL after culturing for 6 h (Fig. 4e). Statistically, the tube junctions, vessel percentage area, and vessel length on bilayer mats were all higher than those on PLCL mats (Fig. 4f–h), indicating the promising potential of bilayer mats in promoting angiogenesis and inspiring optimism for the future of vascular research.

#### 2.2.5 Regulation of bilayer mats on HUASMC phenotype

The phenotypic transformation of VSMCs is a key factor in vascular diseases, including atherosclerosis, aneurysms, restenosis after angioplasty, and diabetic angiopathy. VSMCs maintain the contractile function of blood vessels under normal conditions. However, in some pathological states, VSMCs switch from a contractile phenotype to a synthetic phenotype. This transformation is a significant event in the progression of these diseases. To verify the regulatory effects of bilayer mats, HUASMCs were co-cultured with PLCL and bilayer mats for 24 h, and the expression of related phenotypic marker proteins in the cells was detected. The results show that the contractile gene expression of Calponin in HUASMC increases after treatment with bilayer mats, indicating a potential to restore the normal contractile phenotype of VSMCs. In contrast, the synthetic gene expression of Osteopontin decreases compared with the PLCL mats, suggesting a potential to inhibit the transformation of VSMCs to a synthetic phenotype (Fig. S5). These findings indicate that bilayer mats can inhibit the phenotypic transformation of VSMCs, with significant implications for treating vascular diseases.

#### 2.2.6 Prevention of the infiltration of HUASMCs into the PLCL/SCC layer

During vascular remodeling after vascular injury, VSMCs migrate and deposit in the intima from the media, leading to pathological intimal hyperplasia. Our research has shown that the porosity of the grafts can be regulated by adjusting the flow rates during electrospinning [27]. HUASMCs were incubated on bilayer mats with different pore sizes. For PLCL/KSN//PLCL/SCC (1.8/1.8) mats with a big pore size, many HUASMCs infiltrated from the PLCL/KSN layer to the PLCL/SCC layer (Fig. 4i). For PLCL/KSN//PLCL/SCC (1.8/1) mats with a small pore size, nearly no HUASMCs were observed on the PLCL/SCC layer (Fig. 4j). This is because the pore size of the electrospun mats with a flow rate of 1.8 mL/h is approximately 5.2 μm. In contrast, the pore size for a flow rate of 1.0 mL/h is approximately 1.8 μm. The diameter of contractile smooth muscle cells typically ranges from 5 to 8 μm. A significantly larger pore size in the fibrous mats may result in the infiltration of HUASMCs into the inner layer. In this study, the flow rates of 1.8 mL/h for the outer layer and 1 mL/h for the inner layer were adopted to prepare vascular grafts to inhibit the infiltration of HUASMCs from the outer layer to the inner layer. These results provide reassurance about the potential of bilayer grafts to impede the infiltration of HUASMCs, thereby reducing the risk of intimal hyperplasia.

#### 2.2.7 Gene expression analysis

**Fig. 5:**
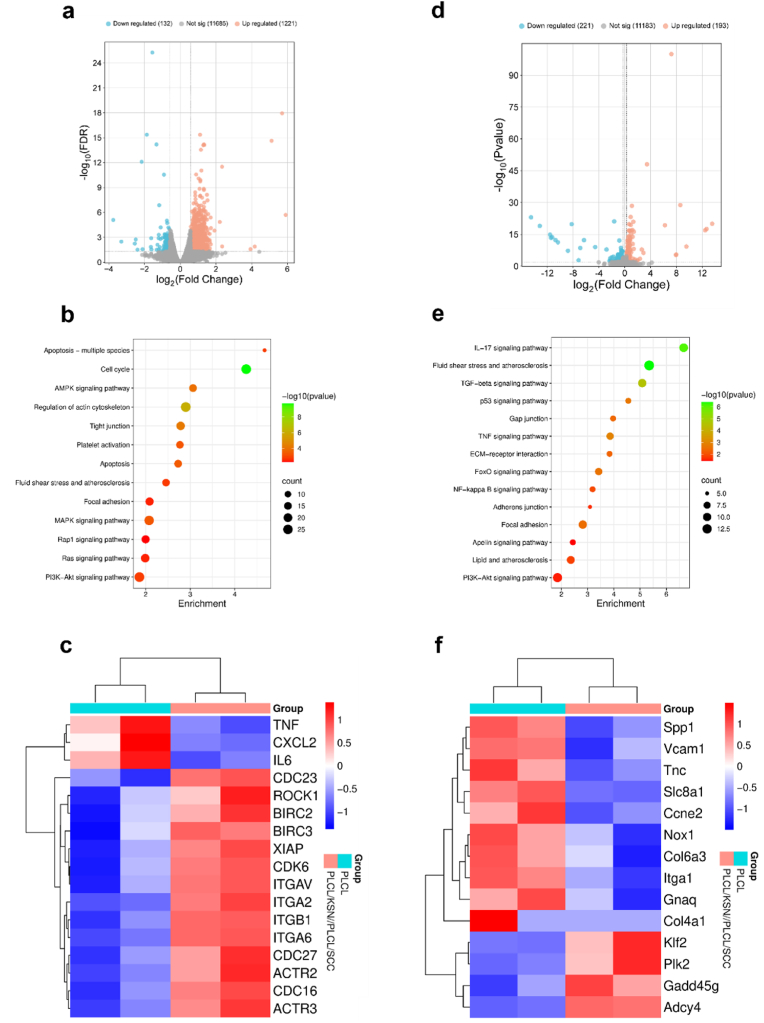
Bilayer mats regulate HUVEC behaviors including (a) Volcano plot showing differentially expressed genes in the PLCL/KSN//PLCL/SCC group compared to the PLCL control group. Downregulated and upregulated genes were colored blue and red, respectively, at significantly differentially expressed thresholds│log_2_FC│>0.58496 and FDR<0.05 (HUVECs); (b) KEGG analysis of differentially expressed genes in the HUVECs. (c) Heatmap of the differentially expressed genes in the PLCL and PLCL/KSN//PLCL/SCC groups; Bilayer mats regulate HUASMC behaviors including (d) Volcano plot showing differentially expressed genes in the PLCL/KSN//PLCL/SCC group compared to the PLCL control group. Downregulated and upregulated genes were colored blue and red at significantly differentially expressed thresholds│log_2_FC│>0.2630344 and p < 0.01; (e) KEGG analysis of differentially expressed genes in HUASMCs; (f) Heatmap of the differentially expressed genes in the PLCL and PLCL/KSN//PLCL/SCC groups.

Based on the above results, the PLCL and PLCL/KSN//PLCL/SCC mats were co-cultured with HUVECs and HUASMCs, respectively. RNA sequencing is tested to further investigate the effects of NO and H_2_S on vascular cells. Rapid endothelialization is crucial for maintaining the patency of SDVGs. It prevents thrombosis, ensures short-term vessel patency, and effectively inhibits excessive proliferation of HUASMCs, thereby preventing vascular stenosis or occlusion. Moreover, mature HUVECs can synthesize and secrete signaling molecules such as NO to regulate vascular contraction and relaxation. The process of endothelialization relies on the proliferation and migration of HUVECs. As shown in Fig. 5a, compared with the PLCL group, 1221 genes were significantly upregulated, and 132 genes were significantly downregulated in the PLCL/KSN//PLCL/SCC group. Enrichment analysis of differentially expressed genes using the KEGG (Kyoto Encyclopedia of Genes and Genomes) database revealed that NO/H_2_S primarily regulates HUVEC adhesion, proliferation, and migration through the PI3K-AKT signaling pathway, focal adhesion, apoptosis, and regulation of actin cytoskeleton (Fig. 5b). Specifically, NO/H_2_S up-regulates the expression of genes such as *ITGAV*, *ITGA2*, *ITGB1*, *ITGA6*, *ACTR2*, *ACTR3,* and *ROCK1* involved in focal adhesion and regulation of actin cytoskeleton, thereby enhancing HUVEC adhesion and migration. By upregulating genes such as *CDC23*, *CDK6*, *CDC27,* and *CDC16* in the PI3K-AKT signaling pathway, NO/H_2_S promoted the proliferation of HUVECs. By upregulating genes such as *BIRC2*, *BIRC3*, *XIAP,* and *PIK3R1* in the apoptosis pathway, NO/H_2_S inhibited the apoptosis of HUVECs**.** These findings suggest that NO/H_2_S plays a significant role in facilitating the rapid endothelialization of vascular grafts. Compared to the bilayer mats group, the PLCL group exhibited a significant up-regulation of genes related to atherosclerosis and inflammation, such as *TNF*, *CXCL2*, and *IL6*, suggesting that the PLCL/KSN//PLCL/SCC group had a positive effect on lipid metabolism and anti-inflammation through the release of NO and H_2_S (Fig. 5c). The PI3K-AKT signaling pathway plays a critical role in angiogenesis. Activation of this pathway promotes endothelial cell proliferation and migration, thereby facilitating angiogenic processes [28,29]. HUVECs were co-cultured with mats and subsequently analyzed using a western blotting. The results demonstrated that phosphorylation levels of PI3K and AKT pathways were significantly increased in the bilayer mat group, indicating that the PI3K-AKT signaling pathway was activated (Fig. S6).

The excessive proliferation of HUASMCs is a critical factor contributing to intimal hyperplasia and restenosis following implantation. Compared with the PLCL control group, 193 genes were significantly up-regulated and 221 genes were significantly down-regulated in the PLCL samples (Fig. 5d). The results revealed that pathways associated with HUASMC proliferation encompassed gap junction, ECM-receptor interaction, adherens junction, focal adhesion, and PI3K-AKT signaling pathway. Additionally, pathways implicated in atherosclerosis comprised fluid shear stress and atherosclerosis, lipid and atherosclerosis and apelin signaling (Fig. 5e). Subsequently, we conducted a comprehensive analysis of the expression levels of relevant genes (Fig. 5f). Specifically, the bilayer mats maintained HUASMC homeostasis by up-regulating *Gadd45g*, *Plk2*, and *Klf2*, while inhibiting cell cycle progression. Down-regulation of *Ccne2*, *Itga1*, and *Tnc* suppressed HUASMC proliferation and migration. In the PLCL group, higher expression levels of *Vcam1* and *Nox1* suggest that the release of NO and H_2_S reduces oxidative stress and inflammatory responses in HUASMCs. Additionally, the bilayer mat group down-regulated *Gnaq* and *Slc8a1*within the apelin signaling pathway, promoting HUASMC relaxation and preventing abnormal proliferation. HUASMCs were co-incubated with mats and then subjected to Western blot analysis. The results showed that the expression of phosphorylated proteins of PI3K and AKT pathways decreased in the bilayer grafts group, and the signaling pathway was significantly inhibited (Fig. S7), which is conducive to suppressing the excessive proliferation of HUASMCs [30]. These findings inspire us with the potential of NO/H_2_S to enhance the rapid endothelialization of vascular grafts, offering a promising outlook for vascular biology.

### 2.3 Anti-oxidant, anti-inflammation, and anti-calcification properties of the bilayer mats

#### 2.3.1 Anti-oxidant properties of bilayer mats

**Fig. 6:**
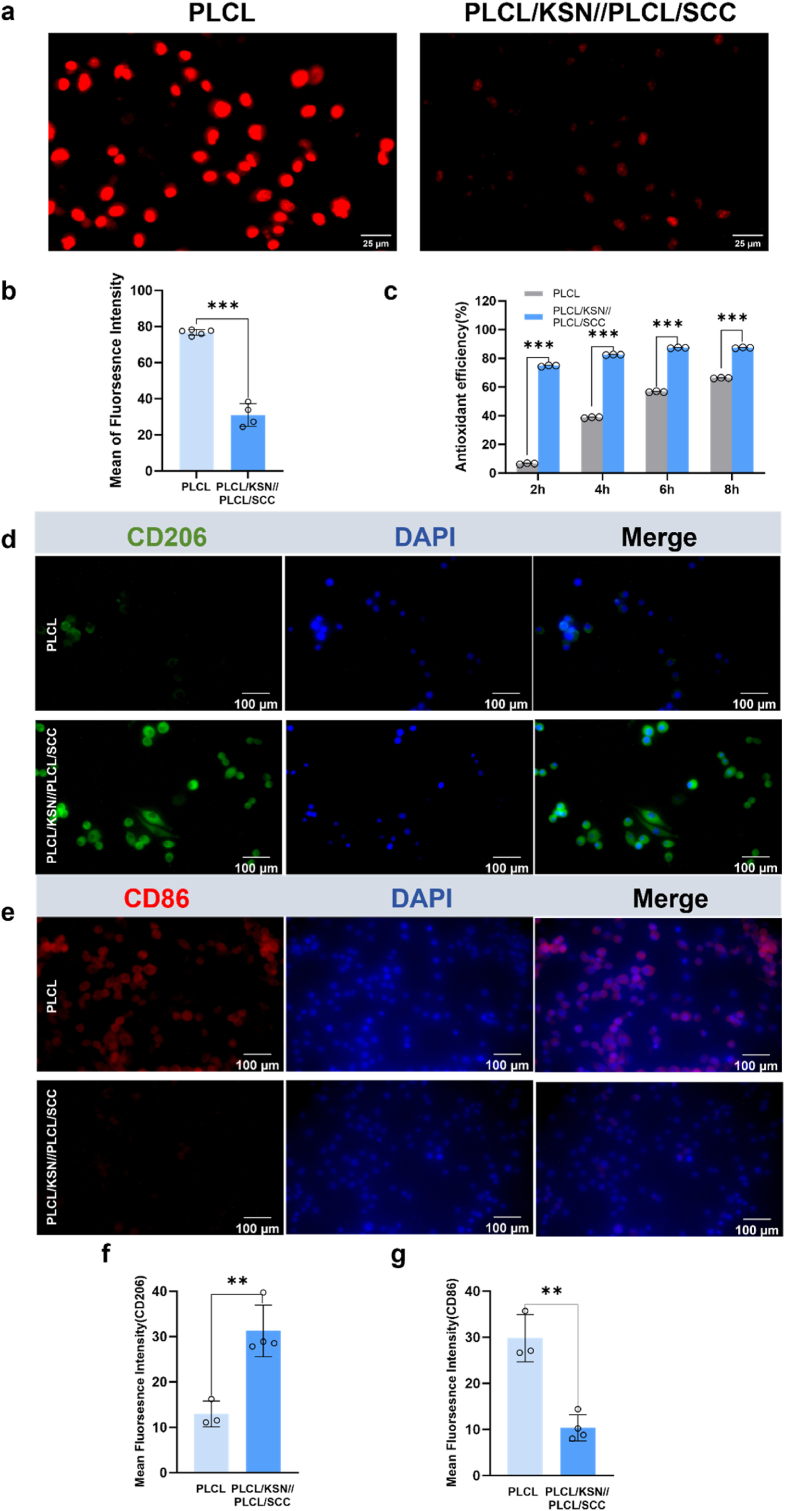
(a) DHE-based fluorescent images of HUVECs after being treated with H_2_O_2_ for 2 h and grown on PLCL and PLCL/KSN//PLCL/SCC mats (+200 μM GSH, +50 μM GSNO); (b) Fluorescence intensity statistics of DHE in HUVECs on PCL and PLCL/KSN//PLCL/SCC mats; (c) DPPH free radical scavenging efficiency of PLCL and PLCL/KSN//PLCL/SCC mats as a function of time; (d,e) Immunofluorescence images of RAW 264.7 macrophages stained with CD206 and CD86 markers on PLCL and PLCL/KSN//PLCL/SCC mats; (f,g) Mean fluorescence intensity of RAW 264.7 macrophages stained with CD206 and CD86 markers. Data are expressed as means ± SD. ∗p < 0.05, ∗∗p < 0.01, and ∗∗∗p < 0.001.

Oxidative stress can lead to lipid peroxidation, induce the expression of adhesion molecules and inflammatory factors, and promote inflammatory cell infiltration, causing vascular endothelial cell dysfunction and damage, as well as contributing to pathological vascular remodeling and atherosclerosis formation [31]. As a natural pigment, SCC exhibits antioxidant activities [32]. It has been found that SCC can modulate oxidative stress and apoptosis in the livers of diabetic mice [18]. H_2_S can enhance the activities of anti-oxidative stress proteases such as glutathione peroxidase (GPX), superoxide dismutase (SOD), and catalase (CAT) to play an anti-oxidative stress function. In this work, the oxidative damage model of HUVECs was established with H_2_O_2_ (400 μM) induction, and the intracellular reactive oxygen species (ROS) content was determined by DHE fluorescence staining. As depicted in Fig. 6a,b, HUVECs exhibited a vigorous fluorescence intensity on PLCL, whereas bilayer mats showed a weak fluorescence intensity. 2,2-Diphenyl-1-picrylhydrazyl (DPPH), a stable free radical scavenger, was used to quantify the antioxidant efficiency of the grafts. Compared with PLCL, the bilayer grafts exhibited higher antioxidant efficiency over time (Fig. 6c)**.** The results demonstrated that the synergistic effect of SCC and H_2_S release endowed the grafts with ROS-scavenging and antioxidant properties, resulting in cell apoptosis and inflammation inhibition.

#### 2.3.2 Anti-inflammation of bilayer mats in vitro

After implantation, the grafts could potentially cause persistent vascular inflammation and accelerate the progression of atherosclerosis. However, our research shows that bilayer grafts have the potential to inhibit inflammation and calcification, thereby offering a promising solution. Macrophages play a crucial role in the progression from the initiation of atherosclerotic lesions to the rupture of atherosclerotic plaques [33,34]. To evaluate the effect of NO and H_2_S release from the bilayer grafts on the inflammatory response, immunofluorescence staining was performed to visualize M1 (CD86 marker) and M2 (CD206 marker) phenotypes, respectively. Compared with PLCL, more CD206^+^ macrophages were observable on bilayer grafts with high green fluorescence intensity (Fig. 6d,f). As depicted in Fig. 6e,g, the number of CD86^+^ macrophages on PLCL was larger than that in the bilayer group, indicating an up-regulated red fluorescence intensity. The results showed that bilayer grafts with dual release of NO and H_2_S polarized more macrophages toward the M2 phenotype, potentially inhibiting inflammation and calcification.

#### 2.3.3 Anti-calcification of bilayer grafts

**Fig. 7:**
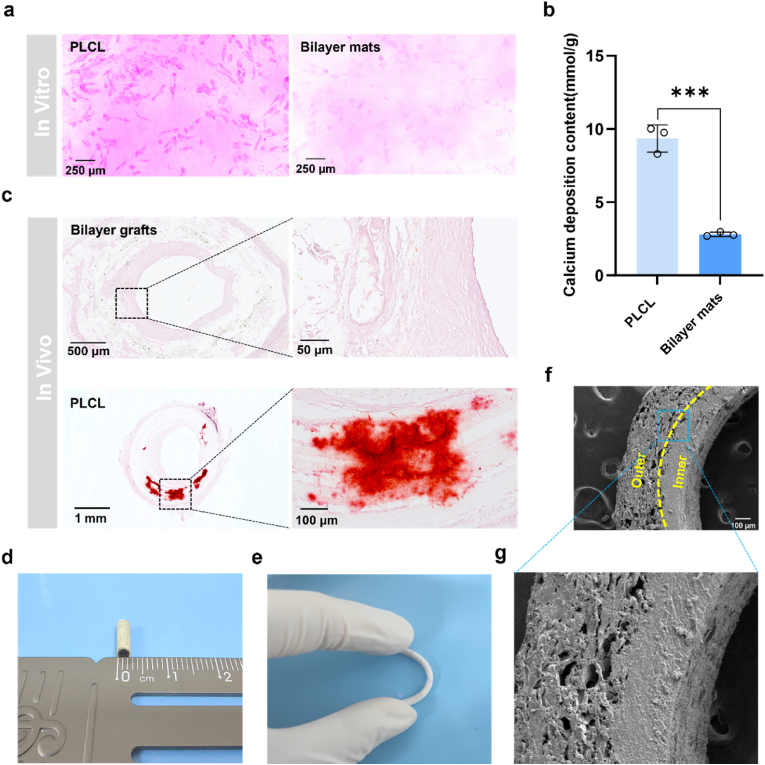
(a) Optical microscope images of HUASMCs stained with Alizarin Red S on PLCL and bilayer mats after culturing for 1 week; (b) Calcium deposits of HUASMCs on PLCL and bilayer mats after culturing for 1 week; (c) Calcification deposits by Alizarin Red S stain for the bilayer grafts and PLCL on abdominal aortic replacement model for 1 month; (d) Macroscopic morphology of grafts; (e) Photographs of grafts under a bent state; (f) SEM images of cross-sectional grafts; (g) Higher magnification images of the cross-section. Data are expressed as means ± SD. ∗p < 0.05, ∗∗p < 0.01, and ∗∗∗p < 0.001.

Vascular calcification can contribute to the narrowing and hardening of artery walls, resulting in a loss of smoothness in the blood vessel wall, leading to stenosis and blockage. Importantly, vascular calcification is primarily caused by the differentiation of VSMCs into osteoblastic cells under pathological conditions, synthesizing and secreting bone morphogenetic proteins (BMPs) [35,36]. PLCL and bilayer mats were incubated with HUASMCs in the calcification-inducing medium. After culturing for 7 d, Alizarin Red S staining verifies that many mineralized nodules are formed on PLCL mats as compared to those on bilayer mats (Fig. 7a). Calcium assay kits were also used to examine the calcium deposition content in HUASMCs. The calcium deposition in the PLCL group is 9.36 mmol/g, much higher than that of 2.80 mmol/g in the bilayer mat group (Fig. 7b). At one month post-implantation, Alizarin Red S staining of graft cross-sections revealed no mineralized nodules in bilayer grafts. In contrast, PLCL grafts showed significant calcium deposits, indicating that the bilayer grafts effectively inhibit calcification (Fig. 7c). The anti-calcification effect of the grafts is mainly attributed to the dual release of NO and H_2_S. It is reported that NO exerts an anti-calcification function by activating the sGC/cGMP pathway or modulating redox-dependent, post-translational modifications, such as S-nitrosylation [37]. It reveals that H_2_S can inhibit the osteoblastic differentiation of valvular interstitial cells (VICs) and affects elastin levels, Stat3 activation, cathepsin S (CAS) activity, and TGF-β1 levels, thereby inhibiting vascular calcification [38,39].

### 2.4 Mechanical properties, hemocompatibility, and biodegradability of bilayer grafts

#### 2.4.1 Mechanical properties of bilayer grafts

The bilayer grafts, as shown in Fig. 7d, exhibit a unique macroscopic morphology and excellent flexibility, allowing them to be bent to suit different implantation requirements (Fig. 7e). The inner and outer layers are seamlessly connected, as evidenced by the SEM images (Fig. 7f). The thicknesses of the outer and inner layers of the grafts are 264 ± 4.47 and 154 ± 7.61 μm, respectively (Fig. S8), further highlighting their unique structure.

As depicted in Fig. S9, the maximum axial and radial tensile strengths of bilayer grafts are 14.73 and 12.12 MPa, respectively. According to the literature, the tensile strength of natural arterial vessels is 3.8 ± 0.4 (axial) and 2.1 ± 0.9 MPa (radial) [40]. Thus, the grafts have excellent mechanical properties for vascular transplantation, providing reassurance about their reliability. The suture retention force of the grafts is approximately 7 N, which is higher than that of the native artery, ensuring that the anastomosis between the grafts and the blood vessel remains intact and prevents blood leakage. Overall, bilayer grafts exhibit excellent mechanical properties, further reinforcing their reliability in vascular transplantation.

#### 2.4.2 Hemocompatibility of bilayer grafts

Blood compatibility is essential for vascular grafts. The hemolysis rates of PLCL, PLCL/SCC, and PLCL/KSN mats are 0.40 %, 0.52 %, and 1.57 %, respectively (Fig. S10a). According to ISO 10993-4 standards, biomaterials with a hemolysis rate of less than 5 % are categorized as non-hemolytic. Thus, bilayer grafts do not cause damage to erythrocytes when in contact with blood in vivo. Platelet activation can trigger the coagulation mechanism, leading to thrombosis. Furthermore, activated platelets are a crucial source of inflammatory mediators, which can induce inflammatory responses in the healthy endothelium and alter normal endothelial functions, thereby accelerating the progression of atherosclerosis [41]. The platelet adhesion on PLCL mats and PLCL/SCC mats is shown in Fig. S10c. More platelets with long and short dendritic activation morphology were found on PLCL mats. Conversely, there was less platelet adhesion and aggregation on PLCL/SCC mats. It is suggested that the PLCL/SCC mats acting as the inner layer of the grafts could prevent platelet activation and maintain the platelet morphology after implantation due to the catalytic generation of NO. NO can increase the activity of guanylate cyclase and promote the production of cyclic guanosine monophosphate (cGMP) in cells, thereby inhibiting ATPase-dependent Ca^2+^ influx and decreasing the cytoplasmic Ca^2+^ concentration in platelets, which suppresses platelet activation and aggregation [42]. The LDH assay was used to quantify the platelets adhered to the mats. As depicted in Fig. S10b, there is no difference between PLCL and PLCL/SCC in the absence of GSNO. The OD value of PLCL/SCC mats in the presence of GSNO is decreased compared to that of PLCL mats, demonstrating that fewer platelets adhere to PLCL/SCC mats. In summary, the PLCL/SCC mats, serving as the inner layer of the grafts, exhibit excellent blood compatibility, thereby inhibiting platelet activation and preventing thrombosis.

#### 2.4.3 Biodegradability of bilayer grafts

Biodegradability is essential for tissue-engineered vascular grafts. The degradation rate of the grafts is related to the structure and constituents of the matrix. In our study, PLCL grafts exhibit a slow degradation rate in vitro, with a weight loss of 8.15 % at 140 d. Regarding bilayer grafts, the degradation rate increases to 17.41 % since keratin can be hydrolyzed to peptides and amino acids by trypsin (Fig. S11). The addition of keratin promotes graft degradation and vascular regeneration.

### 2.5 Evaluation of vascular remodeling in vivo

#### 2.5.1 Patency and luminal morphology

**Fig. 8:**
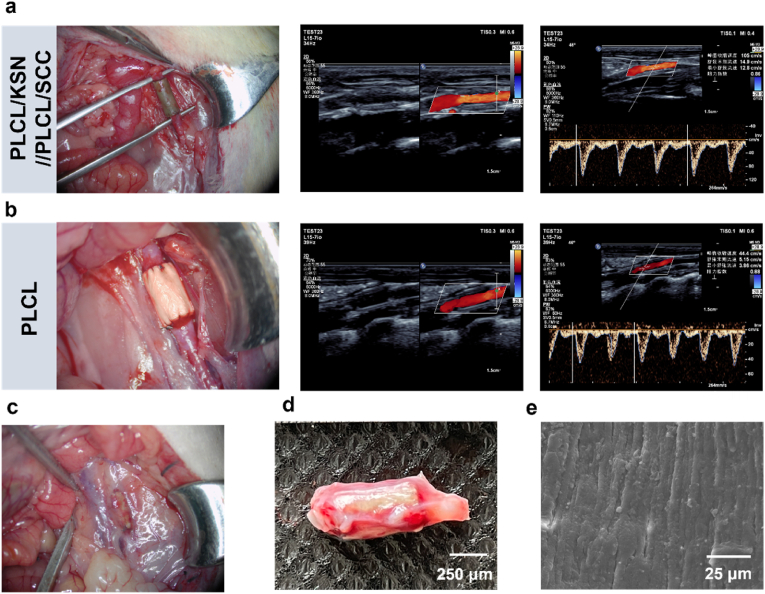
Graft transplantation, ultrasound, macroscopic, and SEM images of the bilayer grafts after one month of transplantation. (a) Re-establishment of the rat carotid artery with the PLCL/KSN//PLCL/SCC grafts and color Doppler ultrasound and lumen diameter of PLCL/KSN//PLCL/SCC grafts at 1 month; (b) Re-establishment of the rat carotid artery with the PLCL grafts and color Doppler ultrasound and lumen diameter of PLCL grafts at 1 month; (c) Perivascular tissue surrounding the PLCL/KSN//PLCL/SCC grafts at 1 month; (d) Macroscopic characteristics of PLCL/KSN//PLCL/SCC grafts at 1 month; (e) SEM images of the inner layer of the PLCL/KSN//PLCL/SCC graft.

Blood pressure in rats is close to that in humans. Thus, a rat abdominal aorta replacement model was used for the in vivo evaluation of bilayer grafts, with PLCL grafts as the control. As depicted in Fig. 8a, the diameter of the PLCL/KSN//PLCL/SCC grafts matches well with that of the rat abdominal aorta. After the blood flow is restored, there is no blood leakage at the anastomosis, and the grafts are well connected to the autologous blood vessel. The autologous blood vessels at the distal end are filled and exhibit regular vascular pulsations, indicating that the transplanted grafts are entirely unobstructed and have good compliance. After surgery for a month, B-mode ultrasound results show that the PLCL/KSN//PLCL/SCC graft lumen is unobstructed, without stenosis or dilation. Doppler ultrasound results indicate that the blood flow signal in the graft lumen is clear. The blood flow velocity and resistance in the grafts are comparable to those in natural blood vessels. The PLCL/KSN//PLCL/SCC grafts exhibit excellent mechanical properties, enabling them to remain stable under high blood pressure. Although the PLCL graft remained patency at one month post-implantation, the blood flow signal was unstable. The flow velocity decreased significantly to 44.4 cm/s, which was considerably lower than that of the natural blood vessel (Fig. 8b). At one month post-transplantation, a rich connective tissue forms around the PLCL/KSN//PLCL/SCC graft, indicating good tissue compatibility (Fig. 8c). Simultaneously, the regular pulsation of the PLCL/KSN//PLCL/SCC grafts can be felt intuitively. It can be observed that the color of the harvested grafts changes from the initial dark green to off-white (Fig. 8d), indicating that the bilayer grafts are gradually degraded for one month, thereby facilitating the remodeling of vascular tissue. The SEM images of the inner surface of the PLCL/KSN//PLCL/SCC grafts reveal that it is entirely covered by new tissue, with no exposed spinning fibers observed, indicating that the vascular intima has completely regenerated (Fig. 8e).

#### 2.5.2 Histological analysis

**Fig. 9:**
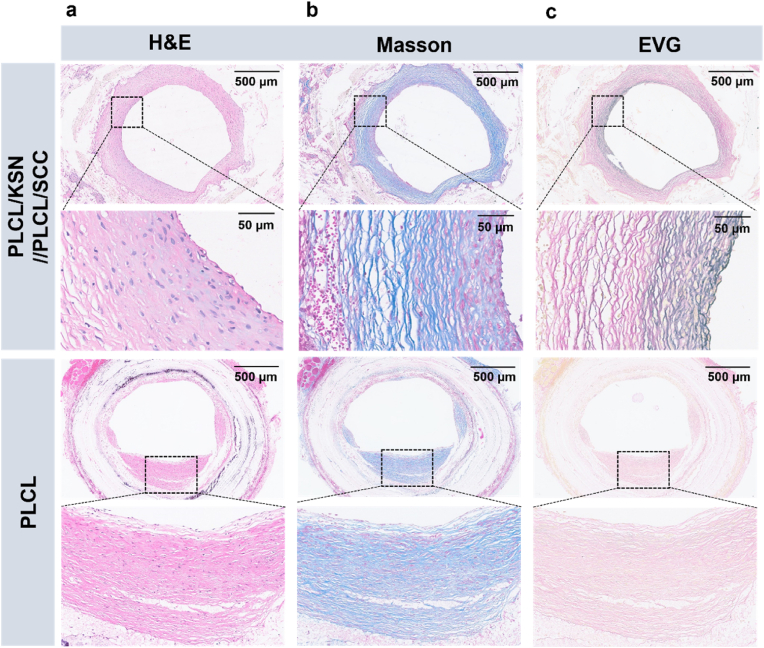
Tissue staining of PLCL/KSN//PLCL/SCC and PLCL grafts. (a) Hematoxylin and eosin (H&E) staining of the cross-sectional grafts; (b) Masson trichrome staining of the cross-sectional grafts; (c) EVG staining of the cross-sectional grafts.

Histological analysis of the transplanted grafts was performed to further understand cellular infiltration and vascular tissue remodeling. From H&E staining, a continuous and thickly layered tissue was observed in the lumen of the PLCL/KSN//PLCL/SCC grafts. In contrast, the PLCL grafts showed no complete neointima in their lumens, with irregular tissue thickness and distribution (Fig. 9a). Furthermore, Masson's trichrome and EVG staining were used to analyze the collagen and elastin components in the grafts, aiming to understand the extracellular matrix (ECM) of the newly formed tissue. Masson's trichrome staining revealed that both PLCL/KSN//PLCL/SCC and PLCL grafts exhibited significant collagen deposition beneath the cellular layer, likely enhancing the compliance and mechanical integrity of the bilayer constructs. However, the middle layer of the PLCL/KSN//PLCL/SCC grafts demonstrated a relatively lower density of collagen fibers (Fig. 9b). Elastic fibers are a key component in maintaining the mechanical properties of the vascular system. Although elastin expression in both types of grafts was limited compared to natural vessels, likely due to the short implantation period, continuous and uniformly distributed black elastic fibers were observed in the lumen of the PLCL/KSN//PLCL/SCC grafts. In contrast, the PLCL grafts exhibited minimal elastin fiber formation (Fig. 9c).

#### 2.5.3 Immunofluorescence analysis of vascular grafts

**Fig. 10:**
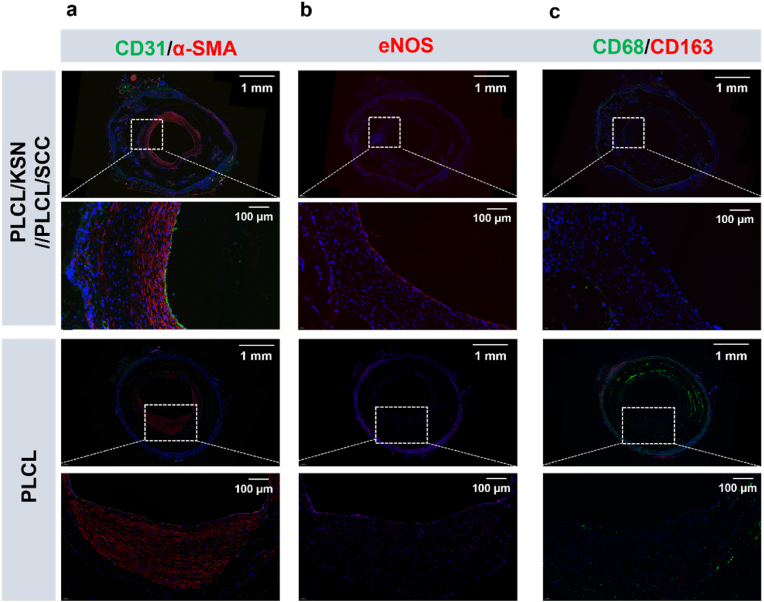
Immunofluorescence images of the cross-sectional grafts; (a) Immunofluorescence staining images of CD31 and α-SMA; (b) Immunofluorescence staining images of eNOS; (c) Immunofluorescence staining images of CD68 and CD163.

Since the endothelium plays a crucial role in both the short-term and long-term patency of vascular grafts, the endothelialization of grafts was analyzed using CD31, a specific marker of endothelial cells. As depicted in Fig. 10a, continuous green fluorescence is observed in the PLCL/KSN//PLCL/SCC graft lumen, indicating that a constant endothelial layer has formed in the vascular grafts after 1 month of transplantation. In contrast, the PLCL grafts exhibited only sparse green fluorescence distribution. To explore the functions of newly born VECs, the expression of the eNOS enzyme in VECs was analyzed through eNOS staining (Fig. 10b). Obviously, compared with the PLCL grafts, the PLCL/KSN//PLCL/SCC grafts exhibited apparent eNOS fluorescence in the EC layer, suggesting enhanced expression of eNOS in these cells. It demonstrates that VECs on grafts can perform related biofunctions by producing NO, such as anti-platelet aggregation activation, leukocyte adhesion inhibition, and inhibition of intimal hyperplasia.

Through α-SMA staining, it was observed that the SMC layer formed by the PLCL/KSN//PLCL/SCC grafts was thinner and more uniformly structured, with better-organized cells, which contribute to maintaining the mechanical integrity of the newly formed blood vessels after graft degradation (Fig. 10a). These findings suggest that the dual release of NO and H_2_S facilitated rapid endothelialization and the development of a well-structured SMC layer, with no significant intimal hyperplasia.

After vascular grafts are implanted, the inevitable inflammatory reaction will profoundly affect the vascular remodeling. Therefore, CD68 and CD163 staining were employed to evaluate the proportion of M2 macrophages, as these markers play a crucial role in the inflammatory response (Fig. 10c). For the neointima on the bilayer grafts, macrophages were nearly absent, suggesting that tissue remodeling had been completed within one month post-implantation. Moreover, a significant presence of M2 macrophages was observed in the tissue surrounding the PLCL grafts, reflecting the persistent tissue remodeling occurring around the graft. The bilayer grafts promote the recruitment of reparative M2 macrophages, which help reduce inflammation and promote tissue regeneration. In contrast, a substantial number of CD68-labeled macrophages were detected within the PLCL graft, indicating a slower degradation rate of the graft and a more significant inflammatory response in the surrounding tissue.

## 3 Discussion

The demand for SDVGs has surged with the rising prevalence of cardiovascular diseases (CVDs). Tissue-engineered SDVGs provide a novel approach to vascular replacement. However, several challenges, including thrombosis, inflammation, restenosis, and late-stage complications, such as intimal hyperplasia, vascular calcification, and late thrombosis, impede vascular reconstruction. Consequently, current study focuses on optimizing the biomimetic structure of tissue-engineered SDVGs to support tissue regeneration more effectively and on enhancing the functionalization of vascular surfaces to regulate the growth behavior of vascular cells [43,44].

Two main strategies for tissue-engineered vascular grafts (TEVGs) are promising: *in vitro* tissue engineering followed by decellularization and in situ tissue engineering. The former strategy involves a complex procedure with a lengthy production time, high costs, and an immune mismatch. The latter strategy only needs a vascular graft, where host cells are recruited in situ to reconstruct new tissue. This in situ strategy may lead to rapid clinical translation. The research on in situ TEVGs focuses on enhancing the microenvironment for vascular regeneration and promoting vascular cell growth by integrating bioactive molecules. NO exhibits various physiological effects, including vasodilation, inhibition of platelet activation and adhesion, suppression of vascular smooth muscle cell proliferation, modulation of stem cell behavior, and regulation of immune response [45,46]. H_2_S exhibits a broad spectrum of physiological functions, including anti-inflammatory, antioxidant, vasodilatory, antifibrotic, and cytoprotective properties. It plays a significant regulatory role in the pathogenesis and progression of cardiovascular diseases such as atherosclerosis, hyperlipidemia, hypertension, and myocardial infarction [13,47]. Recently, the synergistic effects of NO and H_2_S on vascular reconstruction have received more attention. Consequently, combining NO and H_2_S dual gas release strategies for vascular remodeling is expected to construct bionic TEVGs. We have fabricated bilayer grafts where the inner layer with NO generation capability is based on copper (II) complexes and arginine, and the outer layer with H_2_S release ability is based on hydrogen sulfide-releasing keratin and heparin conjugates [[48], [49], [50]]. In addition, we have synthesized S-nitrosylated keratin as an NO donor for vascular grafts [51]. Herein, a novel keratin-based H_2_S donor of KSN was synthesized and incorporated into the outer layer of the grafts to release H_2_S. Additionally, biosafety SCC was introduced into the inner layer for the first time to catalyze the generation of NO. H_2_S synergizes with NO to selectively enhance the adhesion, proliferation, and migration of HUVECs, enabling rapid endothelialization.

Rapid endothelialization on the surface of TEVGs is crucial for vascular regeneration. This process primarily relies on the proliferation and migration of HUVECs, as well as the recruitment, differentiation, and proliferation of circulating endothelial progenitor cells in the bloodstream. Zhu et al. developed a novel H_2_S/NO dual-release molecule, ZYZ-803, which demonstrated significant promotion of EC formation in blood vessels [52]. Our study revealed that the vascular grafts promoted the rapid proliferation and migration of HUVECs by releasing NO and H_2_S, thereby enhancing angiogenesis compared to PLCL (Fig. 3, Fig. 4a) One month at post-transplantation, a continuous and mature endothelial layer had formed in the vascular graft. (Fig. 10a and b). Additionally, mRNA sequencing results indicated that NO/H_2_S regulates endothelial cell adhesion, proliferation, and migration primarily by modulating the PI3K-AKT signaling pathway, focal adhesion, apoptosis, and regulation of actin cytoskeleton (Fig. 5a–c).

VSMCs play a critical role in vascular remodeling because the VSMC layer regulates vascular tone and, together with the ECM, provides vascular tensile strength. However, excessive proliferation of VSMCs can lead to intimal hyperplasia and restenosis. The contractile VSMC layer is essential for maintaining the vascular tone required for long-term graft function and patency. Shvetsova et al. demonstrated that nitric oxide deficiency impairs the phenotypically specific gene expression associated with arterial smooth muscle contraction [53]. Li et al. reported that treatment with NaHS in calcified aorta and carotid arteries prevented smooth muscle cells from transitioning to a diastolic phenotype [54]. In this study, the bilayer grafts effectively inhibit the excessive proliferation and migration of HUASMCs by releasing NO and H_2_S (Fig. 3, Fig. 4b). The contractile gene expression of Calponin in HUASMC increases after treatment with bilayer mats, indicating a potential to restore the normal contractile phenotype of VSMCs (Fig. S5). Animal experiments demonstrate that the thickness of the generated VSMC layer remains uniform and comparable to that of natural blood vessels, thereby maintaining the mechanical integrity of the newly formed vasculature (Fig. 10a). mRNA sequencing results also confirmed the gene signaling pathway identified in the above results (Fig. 5d–f).

Intimal hyperplasia (IH) is a precursor to atherosclerosis, and atherosclerosis lesions are typically associated with inflammation and elevated production of ROS, which impede vascular regeneration. The local inflammatory response and oxidative stress in blood vessels, endothelial dysfunction, and the conversion of VSMC phenotype are the primary triggers for the formation and development of IH. Elevated levels of ROS can promote platelet adhesion and aggregation, trigger the release of inflammatory mediators, compromise EC function, and stimulate the proliferation and migration of VSMCs. The bilayer grafts exhibited superior antioxidant properties, contributing to the release of H_2_S, SCC, and keratins. These reduce oxidative stress damage caused by high levels of ROS in HUVECs (Fig. 6a,b). Furthermore, the bilayer grafts, which concurrently release NO and H_2_S, can promote macrophage polarization towards the M2 phenotype (Fig. 6d–g). Animal studies also demonstrate that the bilayer grafts significantly reduce both the duration and intensity of the inflammatory response (Fig. 10c).

Pore size is vital in tissue regeneration because different cell types require different porous structures. During vascular remodeling, the migration and deposition of HUASMCs from the media to the intima lead to pathological intimal hyperplasia. By adjusting the flow rates for electrospinning, the pore size and orientation of the fibrous mats can be facilely controlled to mimic the extracellular matrix (ECM) structure of natural blood vessels, thereby enhancing cell adhesion and migration [55]. Herein, we fabricated bilayer vascular grafts with gradient porosity by adjusting the flow rate. The larger pore size in the outer layer facilitates the migration and proliferation of HUASMCs, while the smaller pore size in the inner layer effectively prevents their abnormal infiltration. Porous microstructures favor cell ingrowth while decreasing mechanical strength. The balance between mechanical stability and the requisite openness for effective cell recruitment is necessary [56]. The scanning electron microscope (SEM) image of the cross-section of the graft reveals that the pore size of the outer layer is significantly larger than the inner layer (Fig. 7f). The small pore sizes of the inner layer restrain HUASMC migration and infiltration towards the inner layer (Fig. 4i, j).

Vascular calcification represents a critical pathological mechanism that significantly impacts the long-term functionality of vascular grafts. This pathological process is characterized by the abnormal deposits of calcium hydroxyapatite on the graft wall [57], which subsequently induces ectopic calcification within the vascular lumen [58,59]. Such calcification compromises the structural integrity of the vascular wall, leading to increased surface roughness and brittle alterations in mechanical properties. From a histological localization perspective, vascular calcification can be categorized into two primary types: intimal calcification, typically associated with atherosclerotic lipid infiltration and chronic inflammatory responses, and medial calcification, which is predominantly driven by the osteogenic differentiation of VSMCs [60]. This specific cell phenotypic transformation occurs due to the dysregulation of endogenous signaling pathways in VSMCs. PLCL has significant advantages in vascular tissue engineering due to its excellent mechanical properties, including compliance and elastic modulus [61]. However, the high-density ester bond structures within its main chain may lead to excessively rapid hydrolytic degradation. Additionally, the carboxylic groups generated during its metabolic process may facilitate the migration and deposition of calcium ions through charge interactions, potentially inducing ectopic mineralization reactions [62]. Bilayer grafts were functionally modified to enable the surface of PLCL to release NO and H_2_S. Compared to the control group, the bilayer grafts significantly reduced calcified nodules and calcium content, inhibiting vascular calcification (Fig. 7a–c).

Fabrication of tissue-engineered SDVGs by electrospinning offers a facile strategy for vascular replacement. By mimicking the structure of native blood vessels and further incorporating bioactive substances, the vascular regeneration of SDVGs can be significantly enhanced. However, vascular regeneration is influenced by multiple factors such as the graft length, animal size, and health condition. Therefore, it is imperative to optimize further the structural design and compositional elements of vascular grafts to promote neovascularization and address diverse clinical requirements more effectively.

## 4 Conclusion

Bilayer grafts with gradient pore sizes and separated releasing NO and H_2_S were fabricated. The biosafety SCC was introduced, and the novel keratin-based donor of KSN was synthesized for the first time. The bilayer grafts enable rapid endothelialization by selectively enhancing the adhesion, proliferation, and migration of VECs while inhibiting those of VSMCs. More importantly, the released NO and H_2_S synergistically promote the anti-thrombotic, anti-inflammatory, and anti-calcification properties of the grafts. Furthermore, the bilayer grafts synergistically maintained the contractile phenotype of VSMCs and polarized macrophages to the M2 phenotype. The bilayer grafts achieved patency with negligible intimal hyperplasia and inconspicuous calcification in the rat abdominal aorta replacement models for 1 month of implantation. RNA sequencing was conducted to elucidate the potential mechanism of vascular tissue remodeling. The study presents novel candidates for tissue-engineered SDVGs that exhibit rapid endothelialization, antithrombogenicity, anti-inflammation, and anti-calcification properties.

## 5 Experimental section

### 5.1 Materials

β-Mercaptoethanol, tris(2-carboxyethyl)phosphine, 5,5′-dithiobis(2-nitrobenzoic acid), hydroxylamine-O-sulfonic acid, and hexafluoroisopropanol were obtained from Aladdin Chemical Co., Ltd., China. Poly(L-lactide-co-ε-caprolactone)(PLCL) was purchased from Daigang Biomaterial Co., Ltd., China. Cell Counting Kit-8 (CCK-8) was purchased from Boster Biological Technology Co., Ltd., China. Lactate dehydrogenase kits (LDH Cytotoxicity Assay Kit), Alexa Fluor 594-labeled Actin-Tracker Red, Dihydroethidium, Alizarin Red S staining solution, CD206 Mouse Monoclonal Antibody, CD86 Mouse Monoclonal Antibody, Alexa Fluor 488-labeled Goat Anti-Rabbit IgG (H + L), and Cy3-labeled Goat Anti-Rabbit IgG (H + L) were purchased from Beyotime Biotechnology, China. Dulbecco's Modified Eagle's Medium/Nutrient Mixture F-12 (DMEM F-12), Roswell Park Memorial Institute 1640 (RPMI 1640), and fetal bovine serum (FBS) were provided by Hyclone Laboratories. All other chemicals were of analytical grade and used without further purification.

### 5.2 Synthesis of keratin-based H_2_S donor (KSN)

Human hair keratin was extracted by β-mercaptoethanol at 70 °C for 24 h in our lab. Subsequently, keratin solution was transferred to a dialysis bag (3500D) and dialyzed using distilled water containing tris(2-carboxyethyl)phosphine (TCEP, 25 mM) for 48 h. The water was changed every 12 h. At the end of dialysis, the thiol content was determined using Ellman's reagent, a 5,5′-dithiobis (2-nitrobenzoic acid) (DTNB) solution at 410 nm. Then, both KOH and hydroxylamine-O-sulfonic acid at molar ratios of three to thiols were successively added with stirring. After reaction for 12 h under N_2_ protection, the solution was dialyzed and freeze-dried to obtain a keratin hydrogen sulfide donor (denoted as KSN).

### 5.3 Preparation of single-layer mats and bilayer grafts

#### 5.3.1 Preparation of monolayer mats

KSN and sodium copper chlorophyllin (SCC) were mixed with PLCL at a mass ratio of 1:9 and dissolved in hexafluoroisopropanol (HFIP) to form spinning solutions at a concentration of 8 %, respectively. PLCL/KSN mats were fabricated by electrospinning at a flow rate of 1.8 mL/h, while PLCL/SCC mats were prepared at a flow rate of 1 mL/h.

#### 5.3.2 Preparation of bilayer grafts

The electrospinning parameters for the PLCL/KSN//PLCL/SCC grafts are as follows: the voltage is 20 kV, a flow rate of 1.0 mL/h for the PLCL/SCC inner layer, and a flow rate of 1.8 mL/h for the PLCL/KSN outer layer. A round metal rod with a diameter of 2 mm was used to fabricate tubular grafts at 1500 rpm. The distance between the metal rod and the needle is 15 cm. Subsequently, the fabricated PLCL/KSN//PLCL/SCC grafts were subjected to ultrasonic treatment for 5 min to achieve overall pore expansion.

### 5.4 Characterization

Pore diameter of the mats was measured using scanning electron microscopy (SEM, JEOL 6500) by selecting 10 random sites at 500x magnification, and the results are presented as the mean ± standard deviation [63]. The porosities of the inner and outer layers of the grafts were measured using an ethanol immersion method [64]. The chemical compositions of the inner and outer layers of the grafts were tested by X-ray photoelectron spectroscopy (XPS, UIVAC-PHI, Japan) spectra. The release of copper ions was quantitatively determined using inductively coupled plasma (ICP) analysis. The hydrophilicity of the inner and outer layers of the grafts was determined by the sessile drop method with a contact angle measurement (DSA30S, KRUSS GmbH). The axial and circular tensile strengths of the grafts, as well as the suture retention, were measured using a mechanical testing machine. The cross-sectional images of grafts were characterized by SEM, and the thickness of the graft was calculated using ImageJ software.

### 5.5 NO and H_2_S release from mats

#### 5.5.1 Catalytic release of NO from PLCL/SCC mats

The catalytic rate of NO release from the inner layer was tested using S-nitrosoglutathione (GSNO) [65]. Briefly, 50 mg PLCL and PLCL/SCC mats were soaked in 2 mL of GSNO solution (50 μM) and placed at 37 °C in a shaker. The resultant solution was collected and reacted with Griess reagent at specific intervals. The OD value was measured at 540 nm using a microplate reader, and the concentration of NO was subsequently calculated based on the standard curve.

#### 5.5.2 H_2_S generation from PLCL/KSN mats

PLCL/KSN mats (10 mg) were incubated in 5 mL of a GSH solution (pH = 8, 1 mM) with a silver sulfide electrode to test H_2_S release [65]. The voltage value was recorded automatically every 10 min, and the release amount of H_2_S was calculated using the standard curve.

### 5.6 The effect of NO on HUVECs for PLCL/SCC mats

#### 5.6.1 Proliferation and migration of HUVECs

PLCL and PLCL/SCC mats were cut into 1.4 cm discs and placed in a 24-well plate for sterilization with ultraviolet radiation. HUVECs were digested with 0.25 % trypsin and diluted to 1 × 10^4^ cells/mL with RPMI 1640 medium (20 % fetal bovine serum, 1 % penicillin-streptomycin). 1 mL of cell suspension was added to each well and cultured at 37 °C in a 5 % CO_2_ incubator for 72 h. The GSNO solution was added to experimental groups at a final concentration of 50 μM. After incubation, 1 mL of fresh cell culture medium containing 100 μL of CCK-8 solution was replaced per well, and the cells were continued to culture in the incubator for 2 h. Then, 100 μL of solution from each well was transferred to a 96-well plate, and the optical density (OD) value at 450 nm was determined using a Microplate Reader. HUVECs were stained with Cell Tracker Green CMFDA dye for 1 h and washed thrice with PBS. Then, HUVECs were digested with 0.25 % trypsin to a concentration of 5 × 10^5^ cells/mL. PLCL and PLCL/SCC mats were cut into 2.5 cm × 1.5 cm square slices and placed in a 6-well culture plate. A stainless-steel block with a width of 0.5 mm was placed on the surface of the mats, and the 2 mL cell suspension was added to each well. After incubation for 4 h, the block was removed, and the initial position of the cells on the surface of the mats was recorded. A certain amount of GSNO solution (50 μM) was added to the culture medium. After 24 h of culture, the final state of the cells was recorded with a fluorescence microscope, and the migration distance of the cells was analyzed using ImageJ software.

#### 5.6.2 F-actin of HUVECs by immunofluorescent staining

F-actin in HUVECs is visualized by immunofluorescent staining to reveal the cytoskeleton structure, which is crucial for understanding their morphology and function. HUVECs were seeded in 24-well plates at a density of 1 × 10^4^ cells/mL. After cell adhesion, PLCL and PLCL/KSN//PLCL/SCC mats were added to the plate, respectively. The GSNO solution was added at a final concentration of 50 μM. After incubation for 24 h, the cells were washed with PBS three times, then fixed with 1 % glutaraldehyde for 30 min. The cells were repeatedly washed four times for 5 min with 0.1 % X-100 Triton. The Alexa Fluor 594-labeled Actin-Tracker Red dye was diluted with the mixed solution containing 3 % BSA and 0.1 % Triton X-100 at a ratio of 1:100 and added to each well in the dark for 1.5 h. After staining, the cells were rinsed with 0.1 % Triton X-100 and stained with DAPI for 5 min. The cytoskeleton structure of HUVECs was observed and recorded by a fluorescence microscope.

#### 5.6.3 Co-culture of HUVECs and HUASMCs

Co-culturing HUVECs and HUASMCs is essential for studying their interactions and the potential effects of the bilayer mats on both cell types. HUVECs and HUASMCs were stained with Cell Tracker Red CMTPX dye and Cell Tracker Green CMFDA dye (1.5 × 10^−5^ M) for 1 h and then digested with 0.25 % trypsin at a density of 1 × 10^4^ cells/mL, respectively. A mixed cell suspension of HUVECs and HUASMCs in the same cell number (1 × 10^4^ cells/well) was seeded on PLCL and PLCL/SCC mats in a 24-well plate, with the culture medium containing RPMI 1640 medium and DMEM F-12 medium (volume ratio 1:1, 1 mL). After 4 h of cell attachment, the initial quantity ratio of HUVECs and HUASMCs was observed by a fluorescence microscope (MSHOT MF53, China). Subsequently, the proportion of cell proliferation was observed and recorded at 24 h.

### 5.7 The effect of H_2_S on HUASMCs for PLCL/KSN mats

The proliferation and migration of HUASMCs on PLCL and PLCL/KSN mats were performed similarly to that described in Section 5.6.1. GSH (200 μM) was used to simulate physiological conditions for inducing the release of H_2_S from the mats [66].

### 5.8 Synergistic effect of NO and H_2_S on vascular cells for bilayer mats

#### 5.8.1 Synergistic effect of NO and H_2_S on HUVECs and HUASMCs

PLCL/KSN//PLCL/SCC bilayer mats were cut into 1.4 cm discs and placed in a 24-well plate. Then, HUVECs and HUASMCs were treated with 0.25 % trypsin, diluted to 1 × 10^4^ cells/mL, and seeded on mats. Single components of GSNO (50 μM), GSH (200 μM), and a mixture of GSNO and GSH were added to the culture medium, respectively. After culturing for 72 h, the cell viabilities of HUVECs and HUASMCs were assessed using the CCK-8 assay.

#### 5.8.2 Effect of H_2_S release on NO secretion in HUVECs

HUVECs at a density of 1 × 10^5^ cells/mL were cultured on PLCL and PLCL/KSN mats in 60-mm cell culture plates, respectively. The concentrations of NO secreted by HUVECs in the culture medium were detected using Griess reagent on days 1, 2, and 3, respectively.

#### 5.8.3 Effect of NO release on H_2_S secretion in HUASMCs

HUASMCs at a density of 1 × 10^5^ cells/mL were cultured with PLCL and PLCL/SCC mats in a cell culture plate (60 mm), respectively. The concentrations of H_2_S secreted by HUASMCs in the culture medium were tested by the methylene blue method on days 1, 2, and 3, respectively.

### 5.9 Tube formation assay for angiogenesis test

100 μL of Matrigel solution (8.63 mg/mL) was added to a 24-well culture plate and incubated at 37 °C for 30 min to form a gel. Subsequently, HUVECs at a density of 1 × 10^5^ cells/mL were seeded and incubated with PLCL/KSN//PLCL/SCC bilayer mats in the presence of GSNO (200 μM) and GSH (50 μM). After culturing for 6 h, the tubular structures were observed with an optical microscope. The tube junctions, vessel percentage area, and vessel length were analyzed using ImageJ software.

### 5.10 Regulation of bilayer mats on HUASMC phenotype

HUASMCs were plated in 6-well plates at a density of 5 × 10^5^ cells/mL and co-cultured with PLCL and PLCL/KSN//PLCL/SCC bilayers for 24 h. After the cells were washed three times with PBS, 300 μL of cell lysate was added to each well, and the lysate was incubated at 4 °C for 5 min. The lysate was then centrifuged at 12000 rpm to obtain the supernatant. Protein concentration in the supernatant was determined using the BCA Protein Concentration Assay kit. A certain amount of protein supernatant was mixed with buffer at a 5:1 ratio and then heated to boiling. Each lane of SDS-PAGE gel was loaded with a 35 μL protein sample, and the voltage was increased to 120 kV for 45 min, at which point the protein reached the boundary between the concentration gel and the separation gel. Subsequently, proteins were transferred to PVDF membranes under ice-cold bath conditions with a constant current of 300 mA for 90 min. After the membrane transfer was completed, the PVDF membrane was washed three times with TBST solution and then blocked in 1 % BSA solution for 2 h at room temperature. The corresponding primary antibodies (β-actin, Osteopontin, and Calponin) were diluted with antibody dilution solution according to the manufacturer's instructions. The washed membrane was cut and placed in the incubation box, where the antibody was added and incubated overnight at 4 °C. The membrane incubated with the primary antibody was washed thrice with phosphate-buffered saline with Tween (PBST) for 5 min each time. The excess liquid was removed by suction with filter paper and placed in the antibody incubation box. The diluted secondary antibody was poured and incubated for 2 h at room temperature. After washing three times with PBST, the developer solution was added dropwise. The exposure of the protein bands was recorded and photographed using a chemiluminescent image analyzer.

### 5.11 Infiltration of HUASMCs

Different electrospinning rates were used to obtain bilayer mats with varying porosity, denoted as 1.8/1 (1.8 mL/h for the outer layer and 1 mL/h for the inner layer) and 1.8/1.8 (1.8 mL/h for both the outer and inner layers). The infiltration of HUASMCs from outside to the inside of bilayer grafts was carried out. HUASMCs (1 × 10^4^ cells/mL) were stained with Cell Tracker green CMFDA dye (1.5 × 10^−5^ M) and seeded on PLCL/KSN//PLCL/SCC, 1.8/1 and 1.8/1.8 mats, respectively. After 24 h, the number of HUASMCs infiltrating the inner layer was recorded using a fluorescence microscope and counted by ImageJ software.

### 5.12 Transcriptome analysis of HUVECs and HUASMCs

HUVECs and HUASMCs were co-cultured with PLCL and PLCL/KSN//PLCL/SCC mats, and then total RNA was extracted. The purity and concentration of RNA were detected by the Onedrop™ spectrophotometer, and the integrity of RNA was detected on agarose gels. The quality of each library was evaluated by quantitative PCR on the Agilent bioanalyzer 2100. RNA was sequenced using the Illumina NovaSeq X Plus platform. The RNA sequencing process was completed by Genepioneer Biotechnologies Co., Ltd. (Nanjing, China). The volcano plot was utilized to visualize differential gene expression across samples. The heatmap facilitated hierarchical bidirectional clustering analysis for comparing gene expression patterns among different genes and samples. KEGG pathway enrichment analysis was also employed to elucidate the signal transduction pathways of differentially expressed genes.

### 5.13 Antioxidant assay of bilayer mats

#### 5.13.1 DPPH free radical scavenging test

50 mg PLCL and PLCL/KSN//PLCL/SCC bilayer mats were soaked in a centrifuge tube containing 3 mL of a 0.1 mM DPPH solution and placed at 37 °C in the dark. The absorbance values of solutions at 517 nm were detected at 2, 4, 6, 8, and 12 h, respectively. The DPPH solution without mats was set as the control group. The antioxidant efficiency was calculated using the following equation: Antioxidant efficiency (%) = (A_0_-A_s_)/A_0_ × 100, where A_0_ and A_s_ are the absorbance of the control and sample group, respectively.

#### 5.13.2 Reactive oxygen species detection in HUVECs

HUVECs were seeded on PLCL and PLCL/KSN//PLCL/SCC bilayer mats at a density of 1 × 10^4^ cells/mL, respectively. After 12 h of incubation, HUVECs were washed with PBS and then incubated with H_2_O_2_ (400 μM) for an additional 2 h. Then, the cell medium in each well was sucked away, and HUVECs on mats were stained with DHE (10 μM) for 30 min in the dark. The fluorescence intensity in HUVECs was recorded using a fluorescence microscope and analyzed using ImageJ software.

### 5.14 Anti-inflammation assessment in vitro for bilayer mats

RAW 264.7 cells (1 × 10^4^ cells/mL) were cultured with PLCL and PLCL/KSN//PLCL/SCC bilayer mats in a 24-well plate. The addition of LPS (100 ng/mL) was used to mimic the inflammatory environment faced by the grafts after implantation in vivo. After incubation for 24 h, the cells were fixed with 1 % glutaraldehyde for 30 min at 4 °C. Then, the cells were washed thrice with PBS and incubated in 0.1 % X-100 Triton for 10 min. Subsequently, the cells were incubated in a 10 % BSA solution for 1 h. After that, the cells were incubated at 4 °C overnight with CD206 Mouse Monoclonal Antibody and CD86 Mouse Monoclonal Antibody to mark the M2 and M1 macrophages, respectively. Then, the cells were incubated with Alexa Fluor 488-labeled Goat Anti-Rabbit IgG(H + L) and Cy3-labeled Goat Anti-Rabbit IgG(H + L) for 2 h at 37 °C, respectively. The cell nuclei were marked with DAPI for 5 min. Cell morphology and fluorescence intensity were recorded and analyzed with a fluorescence microscope and ImageJ software.

### 5.15 Calcification assay in vitro for bilayer mats

The induction of HUASMC calcification was conducted to evaluate the anti-vascular calcification ability of the bilayer grafts. HUASMCs at a density of 5 × 10^4^ cells/mL were incubated with PLCL and PLCL/KSN//PLCL/SCC mats in a calcification-induced medium (DMEM culture medium + 2.2 mM Na_3_PO_4_+ 2.6 mM CaCl_2_) in a 6-well plate, respectively. After incubation for 1 week, the cells were washed thrice with PBS and then fixed with 95 % ethanol for 10 min. Subsequently, the cells were stained using an Alizarin Red S staining solution (0.2 %, pH 8.3) for 30 min. After being washed thrice with distilled water, red calcium nodules in the cells were observed and recorded under an optical microscope.

The calcium deposition on HUASMCs was quantitatively measured using an MTB Microplate calcium assay kit (Jiancheng Bioengineering Institute, Cat# C004-2-1, Nanjing, China) at 610 nm. Similarly, HUASMCs were incubated in a calcification medium for 1 week and then decalcified with 0.6 M HCl at 37 °C for 24 h. The content of calcium deposition in the supernatant was detected using a calcium assay kit. Distilled water and calcium standard solutions (1 M) were used as controls. HUASMCs were further lysed with 200 μL of lysate at 4 °C for 5 min, and the cell lysate was centrifuged at 12000 rpm for 10 min to obtain protein supernatants. Cell protein concentrations were measured using a BCA Protein Assay Kit. The calcium content of HUASMCs in each well was normalized by the protein content (Calcium/mg protein).

### 5.16 Abdominal aorta replacement in rats

PLCL/KSN//PLCL/SCC bilayer grafts were fabricated on a round metal rod (OD: 2 mm) with the corresponding flow rates for the inner and outer layers. The obtained grafts were dried in a vacuum oven for 24 h to remove excess nonvolatile solvent. To better evaluate the actual effectiveness of bilayer grafts in vascular reconstruction, grafts were implanted into the abdominal aorta of rats. All experimental procedures for in vivo evaluation were performed following animal protection institutional guidelines and approved by the Animal Ethics Committee of Shanghai Children's Medical Center, Shanghai Jiao Tong University School of Medicine (Shanghai, China). SD rats (280-300g, n = 4) were purchased from Kevin's Biotechnology Co., Ltd. (Shanghai, China). Briefly, rats were induced to anesthetize with 3 % isoflurane mixed with air and then continuously maintained in anesthesia with a concentration of 1.5 %. After routine abdominal skin preparation and disinfection, a laparotomy was performed along the midline of the abdomen. The intestines were laid out orderly, and the abdominal aorta was exposed and freed from surrounding tissue. An 8 mm long vascular defect was created by clamping the blood vessel with two hemostats. The bilayer grafts (Length: 8 mm, ID: 1.5 mm) were transplanted to the rat's abdominal aorta through an end-to-end anastomosis using 9-0 sutures. After the blood flow was restored, the intestine was retracted, and the abdominal cavity was closed with 3-0 sutures. After the wound was disinfected, the rats were returned to the cage. At one month at post-operation, the M7/M7T diagnostic ultrasound system, combined with the L146S probe, was used for vascular ultrasound imaging to obtain Doppler and B-mode images. Additionally, spectral Doppler waveform analysis was employed to quantify the flow velocity in the grafts.

At one month after transplantation, all rats were euthanized, and the transplanted bilayer grafts were harvested. To evaluate the intimal characteristics of the obtained grafts, the grafts were dehydrated by a graded series of ethanol, dried, and then observed by scanning electron microscopy (SEM) after gold sputtering. Implanted grafts were fixed in 4 % paraformaldehyde at 4 °C for 4 h, embedded in paraffin, and sectioned at a thickness of 10 μm. Cross sections of grafts were then evaluated with hematoxylin and eosin (H&E) and Masson's trichrome staining, respectively. Moreover, the elastic fiber and collagen fiber components of grafts were analyzed using EVG staining. Immunohistochemical staining for CD31 and α-SMA was performed on fixed sections to examine re-endothelialization and smooth muscle regeneration. Immunohistochemical staining for CD68 and CD163 was used to analyze the inflammation in the bilayer grafts. Immunohistochemical staining for eNOS was further used to evaluate the function of HUVECs in the bilayer grafts. For a more accurate assessment of tissue regeneration, all specimens were harvested from the middle of the grafts for staining.

### 5.17 Statistical analysis

All tests were repeated at least three times, and the results are presented as the mean ± standard deviation. Statistical significance was assessed using GraphPad with ANOVA or Student's *t*-test, denoted by ∗p < 0.05, ∗∗p < 0.01, and ∗∗∗p < 0.001.

## Ethics approval and consent to participate

All animal experiments were performed following the protocols evaluated and approved by Shanghai Children's Medical Center Experimental Animal Welfare and Ethics Committee (Ethics Approval Number：N2024056).

## CRediT authorship contribution statement

**Yu Sun:** Writing – original draft, Methodology, Investigation. **Shunqi Hu:** Validation, Software, Investigation. **Lijuan Wang:** Writing – original draft, Visualization, Validation, Investigation. **Fubang Liang:** Visualization, Validation, Software. **Zeyi Zhou:** Visualization, Validation, Resources. **Yuyuan Zhang:** Visualization, Software, Methodology. **Yanjun Pan:** Validation, Supervision. **Jian Shen:** Supervision. **Meng Yin:** Supervision, Resources, Funding acquisition. **Jiang Yuan:** Writing – review & editing, Project administration, Investigation.

## Declaration of competing interest

The authors declare that they have no known competing financial interests or personal relationships that could have appeared to influence the work reported in this paper.
