# Supplementary material for: Bilayer vascular grafts separately loaded with sodium copper chlorophyllin and keratin-based hydrogen sulfide donor with pro-endothelialization, anti-thrombogenicity, anti-inflammation, and anti-calcification properties
Source: Bioact Mater. 2025 Nov 13;57:247–66. doi: 10.1016/j.bioactmat.2025.11.001 (PMC12661995; doi:10.1016/j.bioactmat.2025.11.001)
Supplement: Multimedia component 1 [file mmc1.docx]

**Supporting Information**

Bilayer vascular grafts separately loaded with sodium copper chlorophyllin and keratin-based hydrogen sulfide donor with pro-endothelialization, antithrombogenicity, anti-inflammation, and anti-calcification properties

1. **Experiment**

**1.1** **Hemocompatibility**

**1.1.1** **Hemolysis test**

Fresh rabbit blood was extracted and centrifuged at 3000 rpm to obtain a red blood cell suspension (RBCs). Briefly, PLCL, PLCL/KSN, and PLCL/SCC mats were immersed in 5 mL of RBCs (2%, normal saline). After incubation at 37 °C for 1 h, 100 µL of the supernatant was centrifuged at 1000 rpm for 5 min and then transferred to a 96-well plate. The OD value of the solution at 540 nm was determined using a Microplate Reader to calculate the hemolysis rate. Normal saline and distilled water were used as negative and positive controls, respectively.

**1.1.2 Platelet adhesion and LDH assay**

Fresh rabbit blood was centrifuged at 1500 rpm to obtain platelet-rich plasma (PRP). 200 μL of PRP and 200 μL of GSNO (50 μM in normal saline) were added to PLCL and PLCL/SCC mats and then incubated at 37 °C for 3 h. At the end of the incubation, the mixed solution of PRP and GSNO co-incubated with the mats was aspirated, and 500 μL of a 2.5% glutaraldehyde solution in normal saline was added to each well to fix the platelets at 4 °C for 2 h. Subsequently, the adherent platelets were observed by SEM.

The LDH activity assay was used to quantitatively analyze platelets adhered to the surface of PLCL and PLCL/SCC mats. 300 μL of LDH release reagent was added to each well and incubated at 37 °C for 1 h. Then, the solution was transferred to a 1.5 mL centrifuge tube for centrifugation at 4000 rpm for 10 minutes. A 120 μL supernatant was transferred to a 96-well plate and mixed with 60 μL of LDH monitoring solution. After incubation at 37 °C for 0.5 h, the optical density (OD) value at 490 nm was determined using a Microplate Reader.

**1.2** **Biodegradation of grafts in vitro**

To evaluate the enzyme-mediated degradation of the grafts, PLCL and bilayer grafts were submerged in 1 mg/mL trypsin solution in PBS and incubated at 37 °C. At preset points of time, the grafts were removed, rinsed with distilled water, and then freeze-dried. The weight difference of dried grafts before and after enzyme degradation was calculated, and the degradation curves were plotted over time. The morphology of grafts after degradation was observed with SEM.

**2. Results**


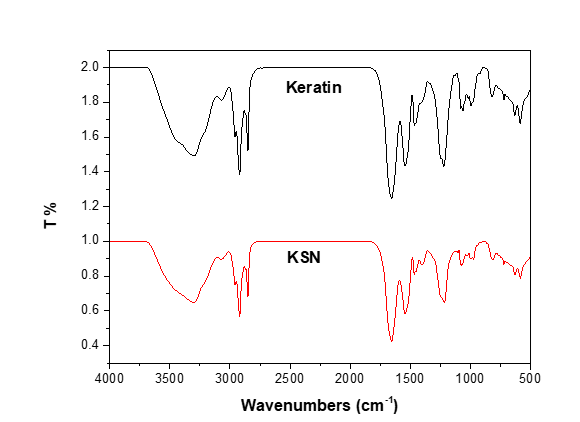


Fig.S1 The FT-IR spectroscopy of Keratin and KSN.


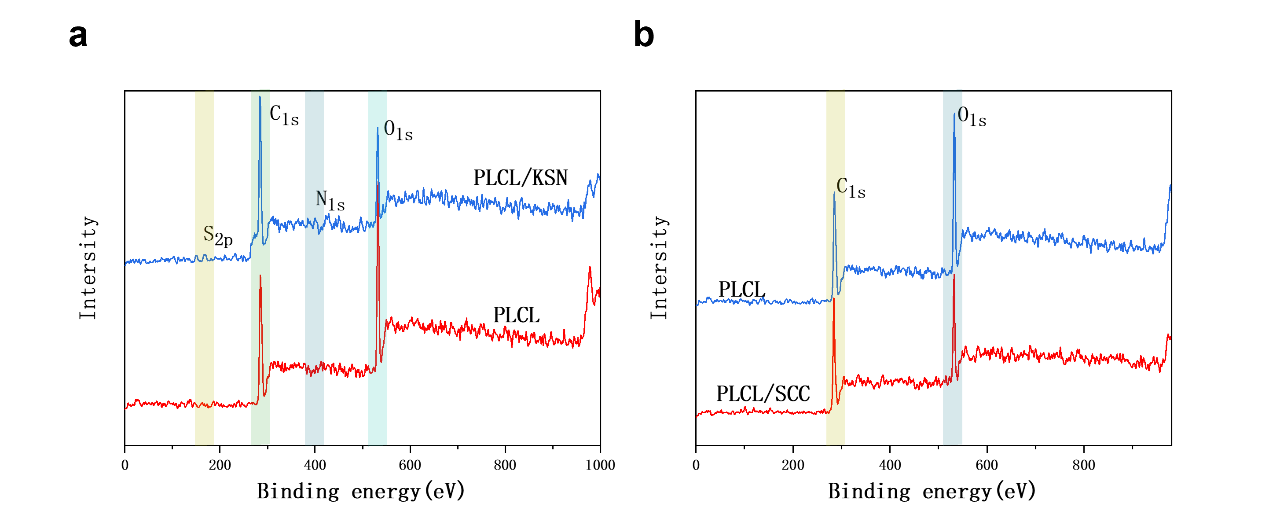


Fig.S2 (a) XPS spectra of PLCL and PLCL/KSN mats; (b) XPS spectra of PLCL and PLCL/SCC mats.


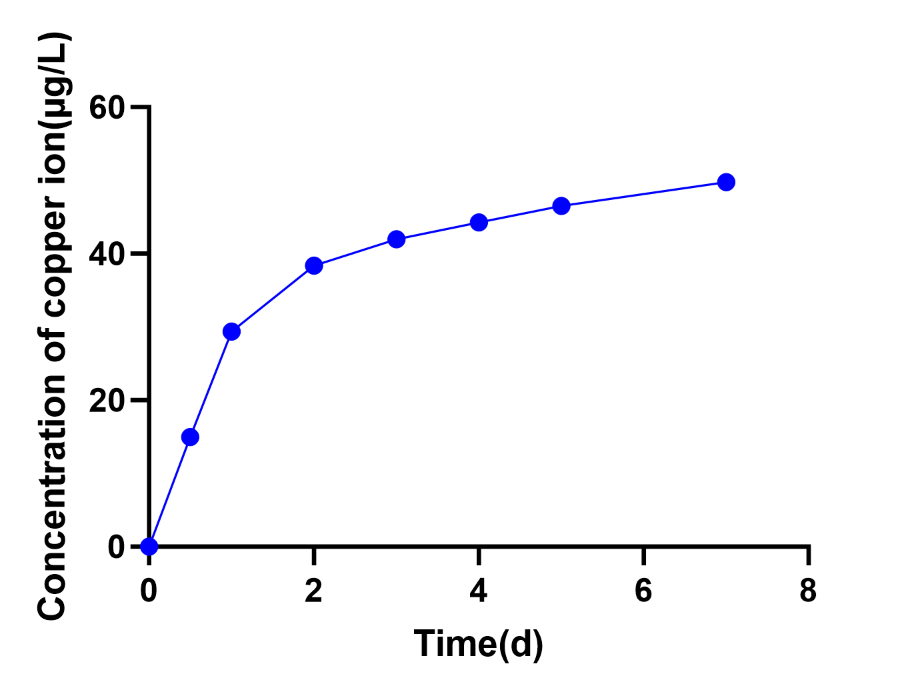


Fig.S3 The release curve of copper ion.


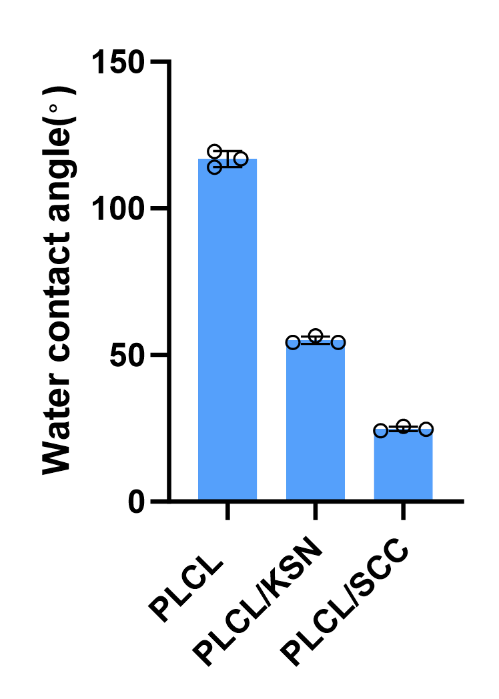


Fig.S4 Water contact angle of PLCL,PLCL/KSN, and PLCL/SCC.


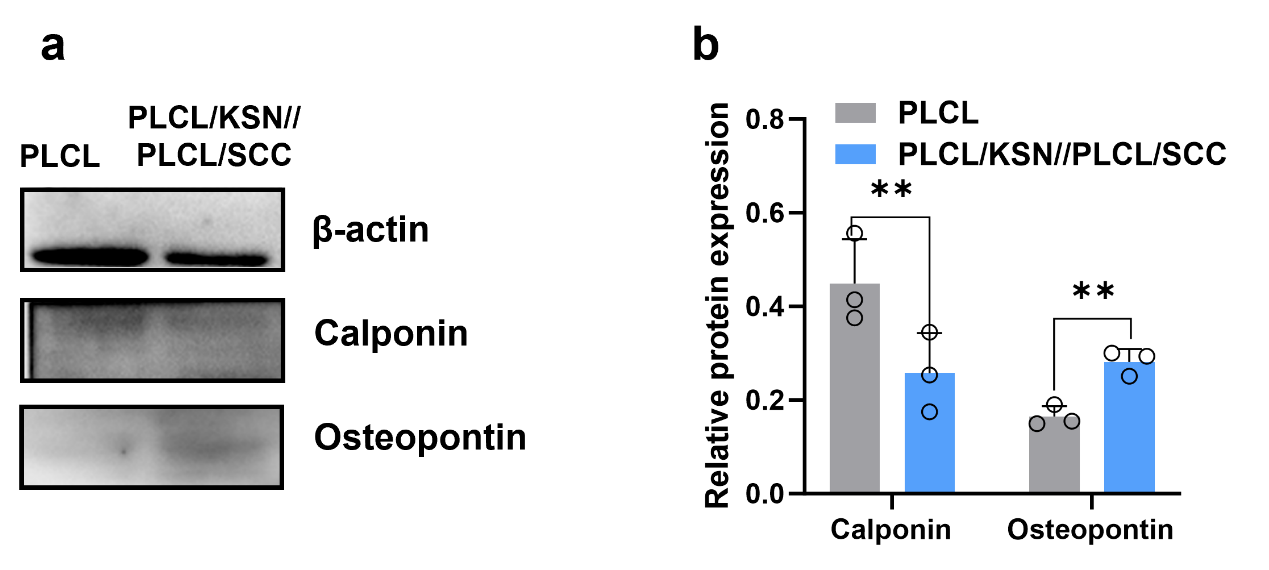


Fig.S5 (a) The expression levels of Calponin, Osteopontin, and β-actin in HUASMCs were detected by Western blotting; (b) Relative expression of Calponin and Osteopontin in HUASMCs.


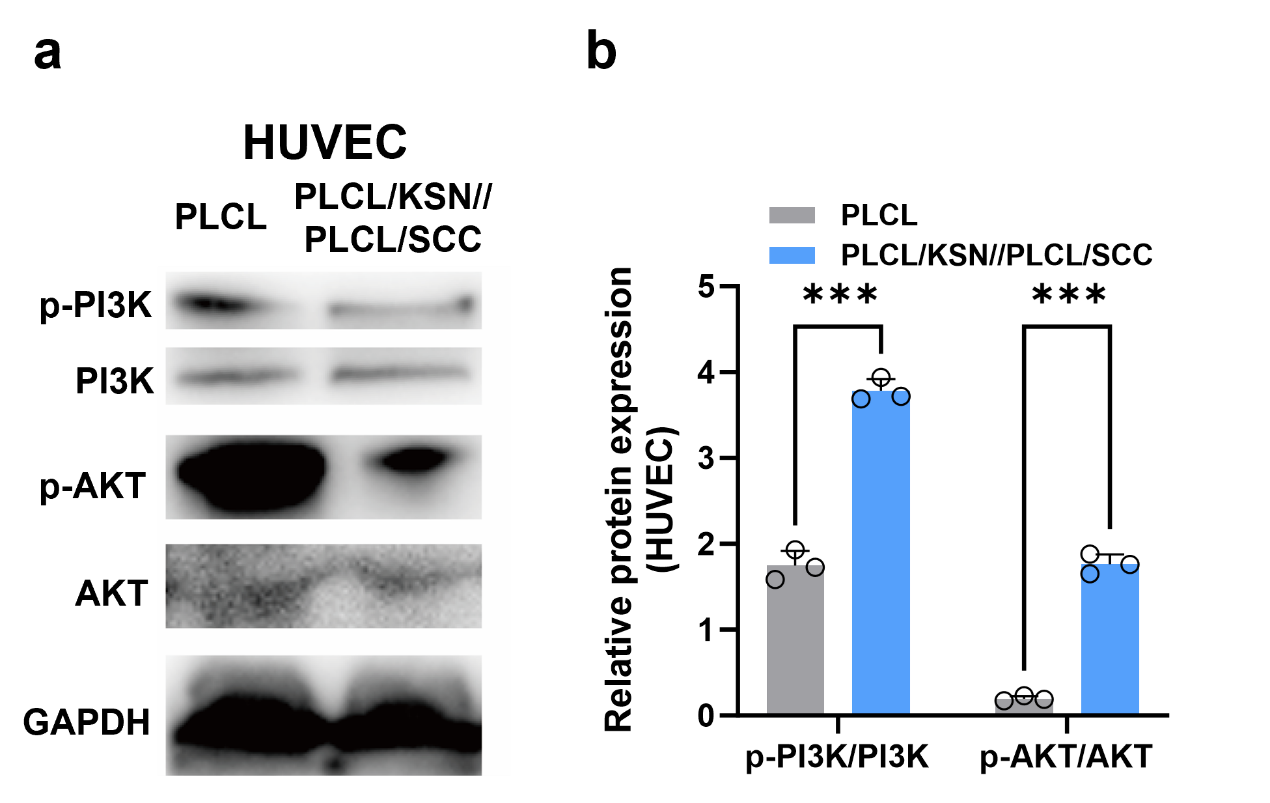


Fig.S6 (a) The expression levels of p-PI3K, PI3K, p-AKT, AKT, and GAPDH in HUVECs were detected by Western blotting; (b) Relative expression of p-PI3K/PI3K and p-AKT/AKT in HUVECs.


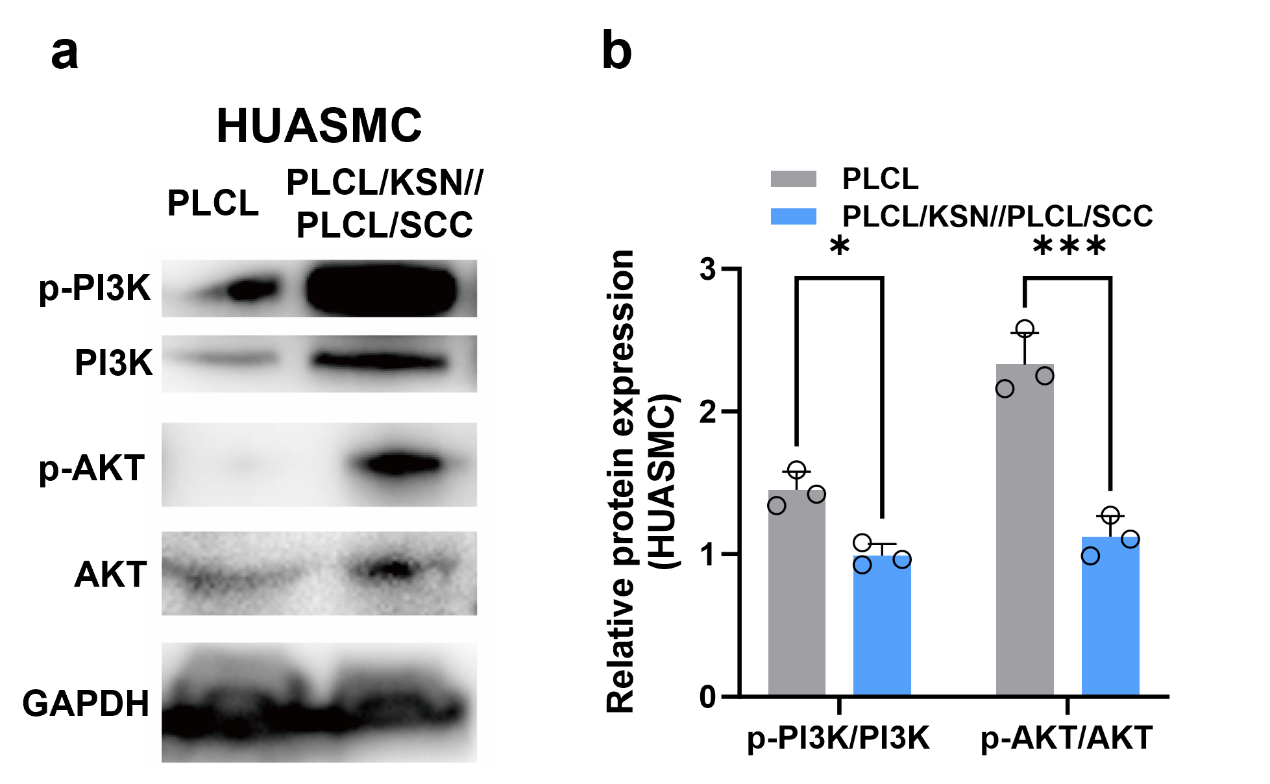


Fig.S7 (a) The expression levels of p-PI3K, PI3K, p-AKT, AKT, and GAPDH in HUASMCs were detected by Western blotting; (b) Relative expression of p-PI3K/PI3K, and p-AKT/AKT in HUASMCs.


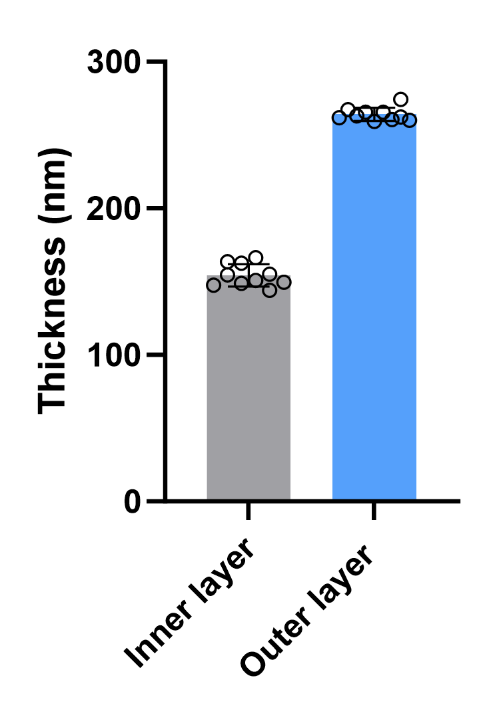


Fig.S8 The thickness of the grafts.


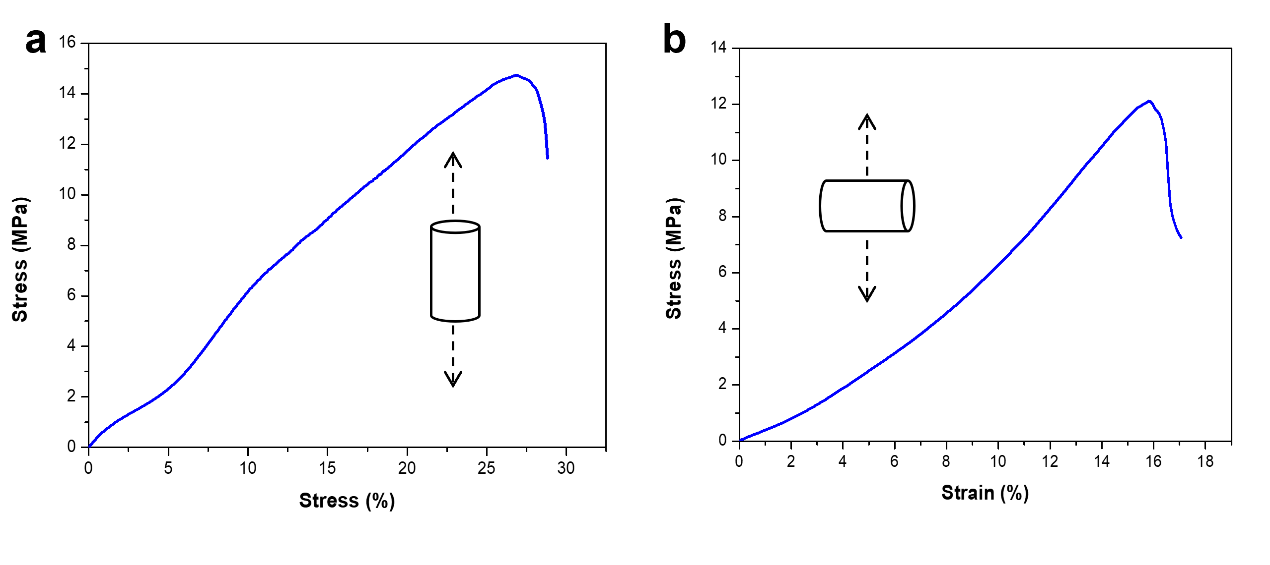


Fig.S9 (a)The axial tensile strengths of bilayer grafts, and (b) the radial tensile strengths of bilayer grafts.


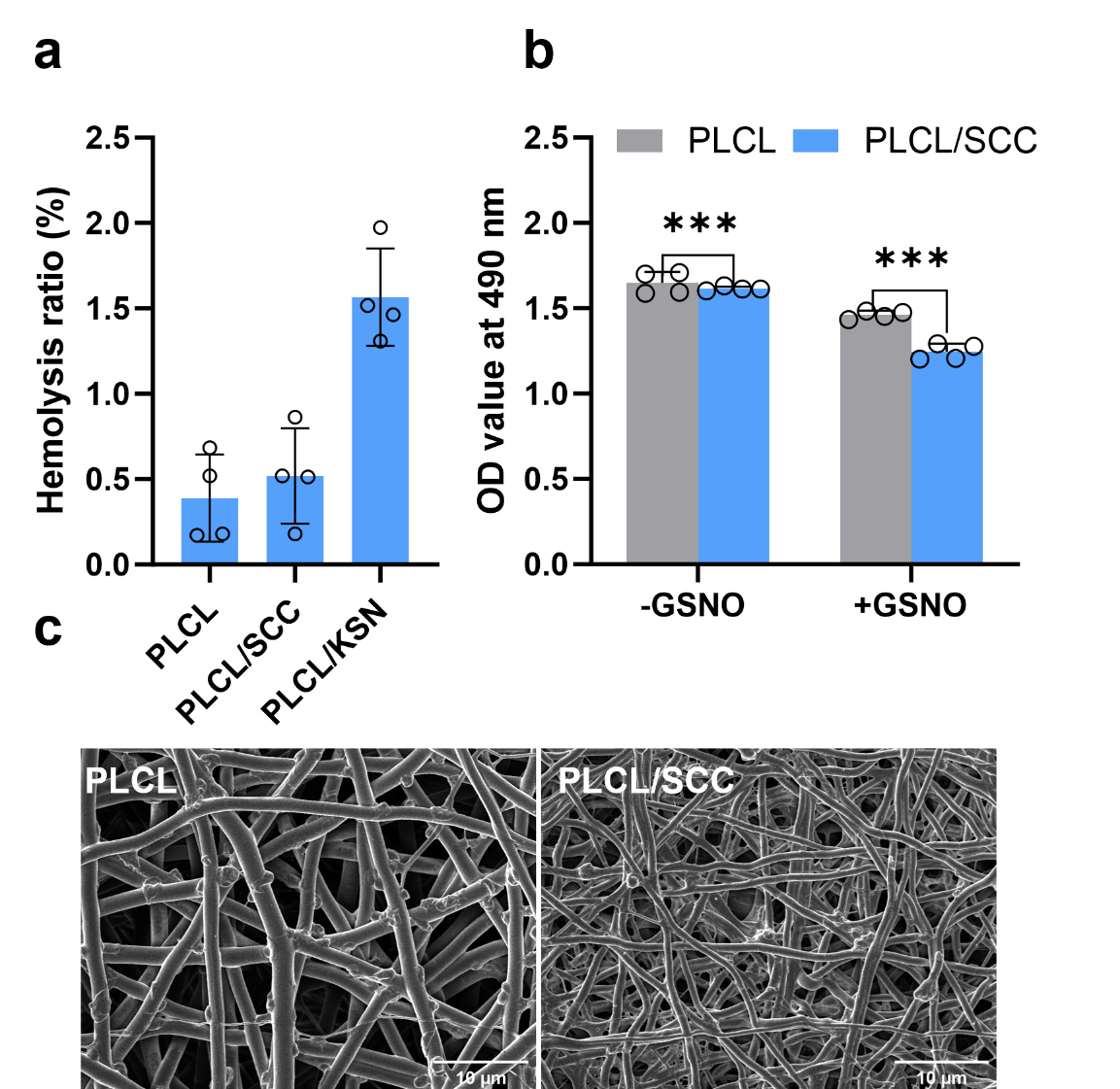


Fig.S10 (a) The hemolysis rates of PLCL, PLCL/SCC, and PLCL/KSN mats; (b) The LDH assay of PLCL, PLCL/SCC mats; and (c) The platelet adhesion on PLCL mats and PLCL/SCC mats.


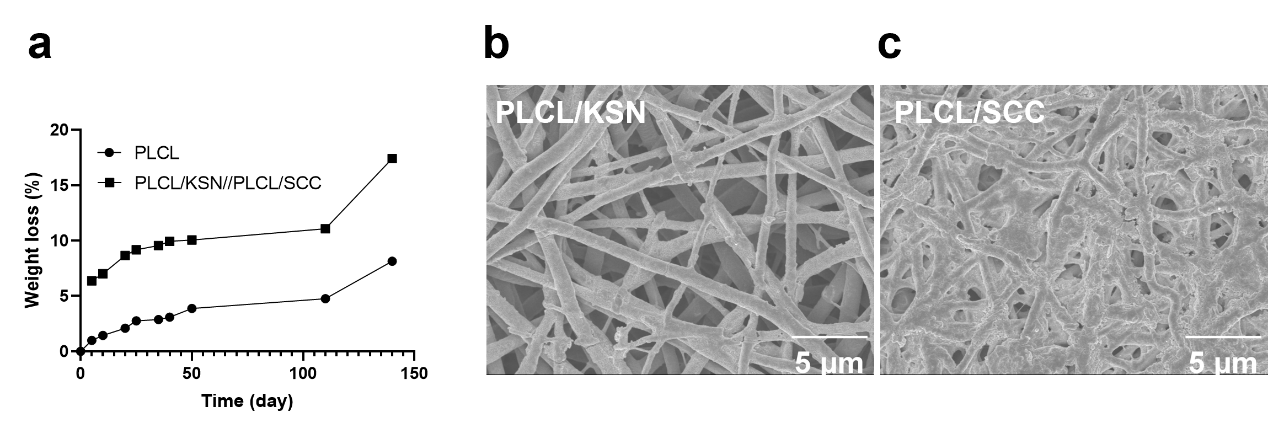


Fig.S11 (a) The degradation rate of PLCL/KSN//PLCL/SC; (b) The SEM of the degraded PLCL/KSN mats; (c) The SEM of the degraded PLCL/SCC mats.
